# Fully coupled hybrid in-silico modeling of atherosclerosis: A multi-scale framework integrating CFD, transport phenomena and agent-based modeling

**DOI:** 10.3389/fbioe.2025.1549104

**Published:** 2025-03-28

**Authors:** Ricardo Caballero, Miguel Ángel Martínez, Estefanía Peña

**Affiliations:** ^1^ Aragón Institute of Engineering Research (I3A), University of Zaragoza, Zaragoza, Spain; ^2^ Biomedical Research Networking Center in Bioengineering, Biomaterials and Nanomedicine (CIBER-BBN), Zaragoza, Spain

**Keywords:** atherosclerosis, agent-based model (ABM), cfd-computational fluid dynamics, transport phenomena, hybrid model

## Abstract

**Introduction:** Atherosclerosis is a complex disease influenced by biological and mechanical factors, leading to plaque formation within arterial walls. Understanding the interplay between hemodynamics, cellular interactions, and biochemical transport is crucial for predicting disease progression and evaluating therapeutic strategies.

**Methods:** We developed a hybrid in-silico model integrating computational fluid dynamics (CFD), mass transport, and agent-based modeling to simulate plaque progression in coronary arteries. The model incorporates key factors such as wall shear stress (WSS), low-density lipoprotein (LDL) filtration, and the interaction between smooth muscle cells (SMCs), cytokines, and extracellular matrix (ECM).

**Results:** Our simulations demonstrate that the integration of CFD, transport phenomena, and agent-based modeling provides a comprehensive framework for predicting atherosclerotic plaque growth. The model successfully captures the mechanobiological interactions driving plaque development and suggests potential mechanisms underlying lesion progression.

**Discussion:** The proposed methodology establishes a foundation for developing computational platforms to test therapeutic interventions, such as anti-inflammatory drugs and lipid-lowering agents, under patient-specific conditions. These findings highlight the potential of hybrid multi-scale in-silico models to advance the understanding of atherosclerosis and support the development of personalized treatment strategies.

## 1 Introduction

Atherosclerosis is a pathology resulting from the accumulation of lipids, inflammatory cells, and extracellular matrix in the arterial wall due to damage in the endothelium, which triggers an immune response leading to the growth and remodeling of the arterial wall (Jebari-Benslaiman et al., 2022; Pederiva et al., 2021). This change in the vessel morphology may result in both blood flow obstruction and outward wall growth that silently develops into a dangerous plaque (Schoenhagen et al., 2001). Atherosclerosis is recognized as the leading cause of cardiovascular diseases (CVDs), accounting for approximately 32% of all global deaths according to the World Health Organization (WHO) (Roth et al., 2020; Wilkins et al., 2017; World Health Organization, 2021). The development of atherosclerosis is a multifactorial process influenced by genetic, environmental, and lifestyle factors, including dyslipidemia, hypertension, smoking, diabetes, and obesity (Pederiva et al., 2021; Poznyak et al., 2022; Martin et al., 2014; Hasheminasabgorji and Jha, 2021; Ito et al., 2019). All these risk factors may affect endothelial cells, increasing their tendency to switch to a mesenchymal-like phenotype, altering their shape, and leading to the appearance of pores in the endothelial barrier (Chen et al., 2015; Yoshimatsu and Watabe, 2022; Esper et al., 2006; Malek et al., 1999).

From a mechanical point of view, disturbed blood flow leads to low wall shear stress (WSS), which is mechano-sensed by endothelial cells, also affecting their shape, turning them into a more circular form, and causing the release of soluble factors that initiate an immune response (Hartman et al., 2021; Caro et al., 1971).

Atherosclerosis starts with the infiltration of low-density lipoprotein (LDL) molecules into the intimal layer of the arterial wall. These LDL molecules undergo oxidation, becoming oxidized LDL molecules (oxLDL), which trigger an inflammatory response. Endothelial cells (ECs) express adhesion molecules attracting circulating monocytes, which migrate to the damaged intima and differentiate into macrophages (MCs). These MCs ingest oxLDL and secrete various soluble factors such as pro-inflammatory cytokines. When the excess of oxLDL surpasses MCs’ absorption capacity, MCs lose most of their cellular functions and are reclassified as foam cells (FCs), which are lipid-laden cells that form the core of the atheromatous plaque (Libby et al., 2019; Lusis, 2000).

As part of the immune response, vascular smooth muscle cells (vSMCs) initially located in the media layer in a contractile phenotype (cSMCs)—inactive and out of the cell cycle, with the sole function of contracting and relaxing to provide tone to the vessel—become activated. They change their phenotype to a synthetic phenotype (sSMCs) and acquire the ability to migrate, proliferate, and produce extracellular matrix (ECM). The continuous accumulation of foam cells and the immune response mediated by sSMCs contribute to the growth of the plaque, leading to arterial wall thickening and luminal narrowing (Gomez and Owens, 2012; Sukhovershin et al., 2016; Nakano-Kurimoto et al., 2009; Bennett et al., 2016).

One of the main clinical complications of atherosclerosis is that the fibrous cap of the plaque may rupture, releasing the lipidic content into the bloodstream, and leading to the formation of a thrombus that can eventually cause a heart attack (Finn et al., 2010; Stefanadis et al., 2017). The mechanisms of plaque rupture have been extensively studied over the years, with the mechanical approach being one of the most common, as the rupture is ultimately a mechanical problem (Peña et al., 2021; Libby, 2013; Virmani et al., 2006; Caballero et al., 2023; Latorre et al., 2023). However, incorporating biological rules that model pathophysiology can make a significant difference in predicting plaque initiation and progression, or explaining patient-to-patient variability caused by emergent phenomena from cell-cell and cell-environment interactions.

Computational modeling has emerged as a powerful tool in atherosclerosis research, providing insights into the disease’s progression and aiding in the development of therapeutic interventions (Çap et al., 2023). Traditional experimental methods, while invaluable, are often limited by ethical, technical, and financial constraints. In contrast, computational models offer a flexible and cost-effective approach to simulate various aspects of atherosclerosis, from molecular and cellular interactions to tissue mechanics.

Several types of *in silico* models have been employed to study atherosclerosis. These include continuous models such as CFD (Ai and Vafai, 2006; Liu et al., 2021; Malvè et al., 2014), FEA (Gu and Bennett, 2022; Tziotziou et al., 2023; Wentzel et al., 2023), transport phenomena (Filipovic et al., 2011; Calvez et al., 2010; Filipović and Kojić, 2004; Olgac et al., 2008; Hernández-López et al., 2021), and discrete models such as agent-based models (ABMs) (Bhui and Hayenga, 2017; Corti et al., 2020).

Continuous models usually solve partial differential equations to describe hemodynamics, the transport of substances within the arterial wall, and the stresses and strains caused by blood pressure. These models are particularly useful for simulating the convection-diffusion-reaction processes that govern the distribution of LDL, oxLDL, and other molecules within the vessel wall.

ABMs, on the other hand, provide a discrete and stochastic framework to simulate the behavior of individual cells. ABMs are well-suited for capturing the heterogeneity and spatial complexity of cellular processes. By representing each cell as an autonomous agent with specific rules of behavior, ABMs can simulate the dynamic and emergent properties of atherosclerotic plaque development due to its bottom-up approach that facilitates the implementation of biological rules inspired by experimental studies.

Recently, the possibility of coupling these two types of models together, combining the strengths of each, has been explored (Corti et al., 2020). These models are known as hybrid models and integrate different multi-scale approaches to capture the main temporal and spatial scales influencing the initiation and progression of atherosclerosis. This merging of continuous and discrete models has shown great potential, providing a more realistic and detailed representation of the plaque.

To the best of our knowledge, there are only a few studies on hybrid models applied to atherosclerosis to date. First, the study developed by Bhui and Hayenga (2017) presented a novel ABM to reproduce the transendothelial migration of leukocytes and the progression of atherosclerosis in coronary arteries, simulating the complex interactions between hemodynamic factors and biological processes. This model used a fixed patch size of 100μm , which, while computationally feasible, might overlook finer spatial variations in substance concentrations, WSS, or the complete intima layer, given that the intima thickness is approximately 10−20 μm . Also Corti et al. (2020) proposed a fully coupled CFD and ABM framework to simulate the development and progression of atheromatous plaque in the superior femoral artery (SFA). Their model provides insights into arterial wall remodeling and plaque formation driven by a disturbed flow, particularly focusing on WSS. However, this study only includes WSS as a trigger for vessel remodeling. This could pose a problem when attempting to incorporate calcifications or therapies that block macrophage activity to reduce inflammation, as some of the key cellular processes involved in the immune response are not modeled. According to our understanding, if the ultimate goal of developing hybrid models is to test different therapies in various scenarios or to explain the variability in patient responses to similar stimuli, it is vital to model the pathophysiology in greater detail. This includes incorporating additional factors such as the transport of plasma and LDL molecules within the wall, the immune response provided by MCs and sSMCs, like sSMCs’ migration and proliferation, or the secretion of growth factors leading to plaque progression.

By enhancing the model to include these elements, hybrid models can more accurately simulate the progression of atherosclerosis and its response to treatments. This comprehensive approach will provide better insights into personalized therapeutic strategies, ultimately improving patient outcomes.

In this work, we present a fully coupled framework that begins with an idealized healthy artery and simulates the development of atheroma plaque under pathological conditions driven by WSS and oxLDL concentration. This hybrid model integrates two different approaches: a continuous approach previously developed by our research group (Hernández-López et al., 2021), which computes hemodynamics and substance transport to determine WSS and oxLDL levels, and a discrete approach, where an ABM responds to the continuous model’s inputs to simulate cellular dynamics.

Specifically, the CFD analysis performed on a 3D idealized coronary artery model provides hemodynamic input to multiple 2D ABMs, which simulate the cellular behavior driving plaque progression. The models are coupled through a semi-automated methodology, reconstructing the 3D vessel from the growth data of 2D cross-sections.

This integration enables a more accurate representation of plaque development by dynamically updating the artery’s geometry as the plaque grows, capturing variations in WSS and oxLDL concentration over time.

Furthermore, a sensitivity analysis focused on the most influential variables is carried out to evaluate how changes in input parameters affect the model’s behavior. This analysis tests the model’s robustness in estimating the progression of atherosclerosis in the coronary artery, providing critical insights into its predictive accuracy.

## 2 Methods

**FIGURE 1:**
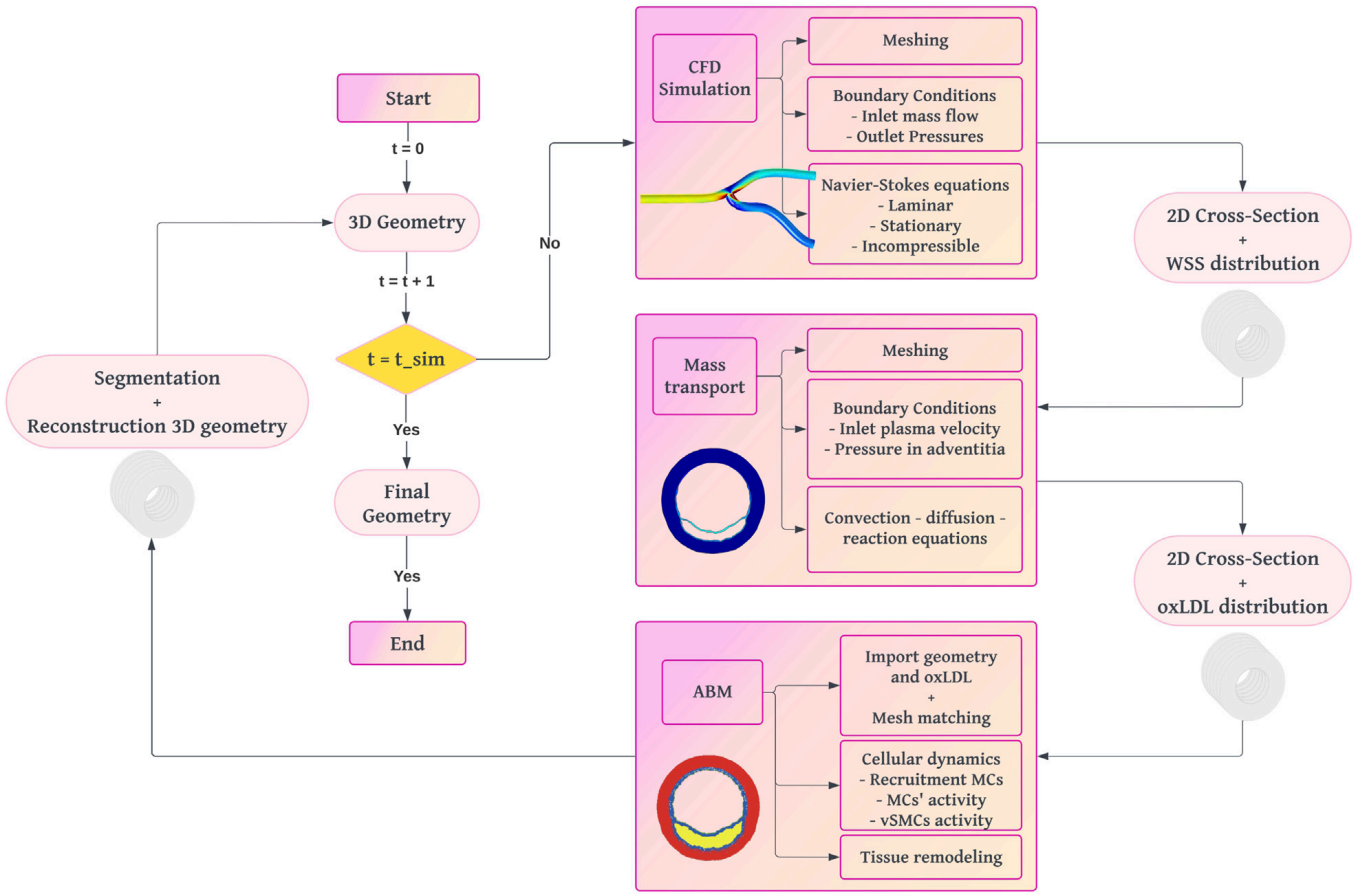
Workflow diagram of the fully coupled multi-scale framework for plaque growth reproduction. The process begins with a 3D geometry of a healthy artery. CFD simulations calculate WSS. Mass transport simulations determine oxLDL distribution. An ABM simulates cellular dynamics to compute plaque growth and vessel remodeling. The iterative process updates the 3D geometry until the final simulation time is reached.

We developed a fully coupled methodology starting from an idealized 3D geometry of a coronary artery, on which we performed a CFD study to obtain the WSS (Figure 1). Using the WSS profile as input, we fed a mass transport model, which predicted the concentration of oxLDL in the wall through convection-diffusion-reaction equations. In areas under higher risk for atherosclerosis due to its oxLDL concentration, we selected five cross-sectional slices where we calculated wall growth using an agent-based model.

### 2.1 3D coronary artery geometry

We built an idealized 3D geometry of the lumen of a healthy coronary artery. The artery diameter was assigned based on data from the literature for different types of coronary arteries (MacAlpin et al., 1973; Dodge Jr et al., 1992; Ohayon et al., 2008). Based on these data, we selected representative constant diameter values of $\phi_{\mathrm{int}}=3.6\text{ mm}$ and $\phi_{\mathrm{ext}}=5.0\text{ mm}$ with a constant wall thickness of 0.7 mm .

### 2.2 CFD-based hemodynamic study

Due to the substantial evidence supporting the role of WSS as a critical factor in the initiation and progression of atherosclerosis (Caro et al., 1971; Liu and Tang, 2010; Filipovic et al., 2013; Olgac et al., 2008; Hartman et al., 2021), which correlates regions of low WSS with areas prone to lipid accumulation, endothelial dysfunction, and subsequent plaque formation, we utilized the WSS as the main trigger of plaque growth in our predictive model. For this purpose, we conducted a CFD study on a 3D idealized geometry of the lumen of a coronary artery using COMSOL Multiphysics 5.6 (COMSOL AB, 2020), solving the Navier-Stokes equations (Equations 1, 2) under the assumptions of laminar, Newtonian, incompressible, and stationary flow. The mesh resolution was carefully chosen based on a mesh sensitivity analysis conducted by our group (Hernández-López et al., 2021), ensuring that the grid resolution was appropriate for accurately capturing the flow dynamics and WSS values.

$$\rho \frac{\partial u_{b}}{\partial t}+\rho_{b}\left(u_{b}\cdot \nabla \right)u_{b}=\nabla \cdot \left[-P_{b}I+\mu_{b}\left(\nabla u_{b}+{\left(\nabla u_{b}\right)}^{T}\right)\right]+F_{b},$$ (1)

$$\rho_{b}\nabla \cdot u_{b}=0,$$ (2)

Boundary conditions included a no-slip condition of blood flow along the endothelium, a specified mass flow rate at the inlet, and pressure conditions at the outlet, following Murray’s law (Murray, 1926). Table 1 summarizes the parameters used in this model.

**TABLE 1:** Blood flow parameters and boundary conditions for CFD study.

Parameter	Description/Boundary condition	Value	References
$\mu_{b}$	Blood dynamic viscosity	0.0035 Pa⋅s	Milnor (1989)
$\rho_{b}$	Blood density	$1050\;{\text{kg/m}}^{3}$	Milnor (1989)

Boundary conditions	Value
Inlet mass flow	$1.603\cdot 10^{-3}\;\text{kg/s}$
Output pressure 1	110 mmHg
Output pressure 2	110 mmHg

### 2.3 Mass transport model

We extracted several cross-sections along with their corresponding WSS data from the 3D idealized geometry used in the CFD model. For each cross-section, we defined the different layers of the vessel wall, incorporating the internal elastic lamina (IEL) as a 20μm offset from the endothelium (Prati et al., 2010). The remaining wall thickness corresponds to the media layer, with the external elastic lamina (EEL) as the outer boundary. The adventitia was not modeled due to its negligible impact on atherosclerosis. Although the endothelium, IEL, and EEL were modeled as a single 2D line in our model their real thicknesses, which are crucial for accurately calculating plasma and LDL filtration, were incorporated into the relevant equations to ensure a realistic representation of the filtration process. The geometrical data of each layer is summarized in Table 2.

**TABLE 2:** Properties of coronary artery layers.

Layer	Thickness (µm)
Endothelium	2.0
Intima	20.0
Internal Elastic Lamina (IEL)	2.0
Media	676.0

In this model, WSS is utilized to compute the concentration of oxLDL along the arterial wall. The mass transport process initiates with the filtration of plasma through the endothelium. This filtration is driven by changes in endothelial porosity, which are directly correlated with the magnitude of WSS. Decreased WSS leads to altered permeability, facilitating the translocation of plasma and substances such as LDL, into the intima (Hartman et al., 2021; Caro et al., 1971). Once LDL molecules penetrate the arterial wall, they undergo oxidation, transforming into oxLDL (Libby et al., 2019; Lusis, 2000). Inside the arterial wall, oxLDL molecules continue to move due to both convective effects, caused by plasma filtration, and diffusive effects, driven by concentration gradients within the wall. This establishes the concentration gradient of oxLDL across the intima layer, setting the stage for further biological interactions within the arterial wall. Our research group previously developed this model (Hernández-López et al., 2021) and it has been adapted for this work. All the parameters needed for the computation of the mass transport model are summarized in Table 3.

**TABLE 3:** Parameters for plasma and LDL flow through the wall.

Parameter	Description	Value	References
Physical Properties			
$k_{\mathrm{int}}$	Intima permeability	$2.2\cdot 10^{-16}\;{\text{m}}^{2}$	Ai and Vafai (2006)
$k_{\mathrm{med}}$	Media permeability	$2.0\cdot 10^{-18}\;{\text{m}}^{2}$	Ai and Vafai (2006)
$\epsilon_{\mathrm{int}}$	Intima porosity	0.983	Ai and Vafai (2006)
$\epsilon_{\mathrm{med}}$	Media porosity	0.258	Ai and Vafai (2006)
$L_{p,snj}$	Normal junction conductivity	$2.0193\cdot 10^{-8}\;\frac{\text{cm}}{\text{s}\cdot \text{mmHg}}$ *	Tedgui and Lever (1984)
$\mu_{p}$	Plasma dynamic viscosity	0.001 Pa⋅s	Milnor (1989)
$\rho_{p}$	Plasma density	$1050\;\frac{\text{kg}}{{\text{m}}^{3}}$	Milnor (1989)
$D_{\mathrm{free}}$	Free LDL diffusion coefficient	$5\cdot 10^{-10}\frac{{\text{m}}^{2}}{\text{s}}$	Dabagh et al. (2009)
$D_{\mathrm{LDL}}$	LDL diffusion coefficient in the wall	$1\cdot 10^{-9}\frac{{\text{m}}^{2}}{\text{s}}$	Prosi et al. (2005)
Dimensions			
$A_{\mathrm{unit}}$	Unit area	$0.64\;{\text{mm}}^{2}$	Olgac et al. (2008)
$l_{ij}$	Length of a leaky junction	2 μm	Weinbaum et al. (1985)
$R_{\mathrm{cell}}$	Endothelial cell radius	15 μm	Weinbaum et al. (1985)
$w_{l}$	Half-width of a leaky junction	20 nm	Weinbaum et al. (1985)
$r_{\mathrm{LDL}}$	LDL radius	11 nm	Tarbell (2003)
Pressures			
$P_{\mathrm{EEL}}$	Pressure at the EEL	30 mmHg	Ai and Vafai (2006)
$\Delta P_{\mathrm{End}}$	Pressure drop in the endothelium	21.6364 mmHg **	Tedgui and Lever (1984)
Concentrations			
$C_{l}$	LDL concentration in the lumen	$4.914\;\frac{\text{mol}}{{\text{m}}^{3}}$	Reiner et al. (2011)

*The value of the parameter is dependent on the considered artery (Table value for coronary arteries).

**The value of the parameter is dependent on the intraluminal pressure (Table value of 110 mmHg).

#### 2.3.1 Plasma filtration

Plasma filtration along the wall was modeled using Darcy’s law (Equation 3), which computes the filtration velocity $\left(u_{p}\right)$ based on the permeability of the medium (K) , the dynamic viscosity of plasma $\left(\mu_{p}\right)$ , and the pressure drop across the medium (∇P) .

$$u_{p}=-\frac{K}{\mu_{p}}\nabla P$$ (3)

We set the plasma velocity filtrating across the endothelium and the pressure at the EEL as boundary conditions.

The flow of plasma across the endothelium was calculated using a modified version of Starling’s law (Equation 28, Supplementray Appendix SA1.), which neglects osmotic pressure (Tedgui and Lever, 1984) (Equation 4).

$$J_{v}=L_{p}\Delta P$$ (4)

**FIGURE 2:**
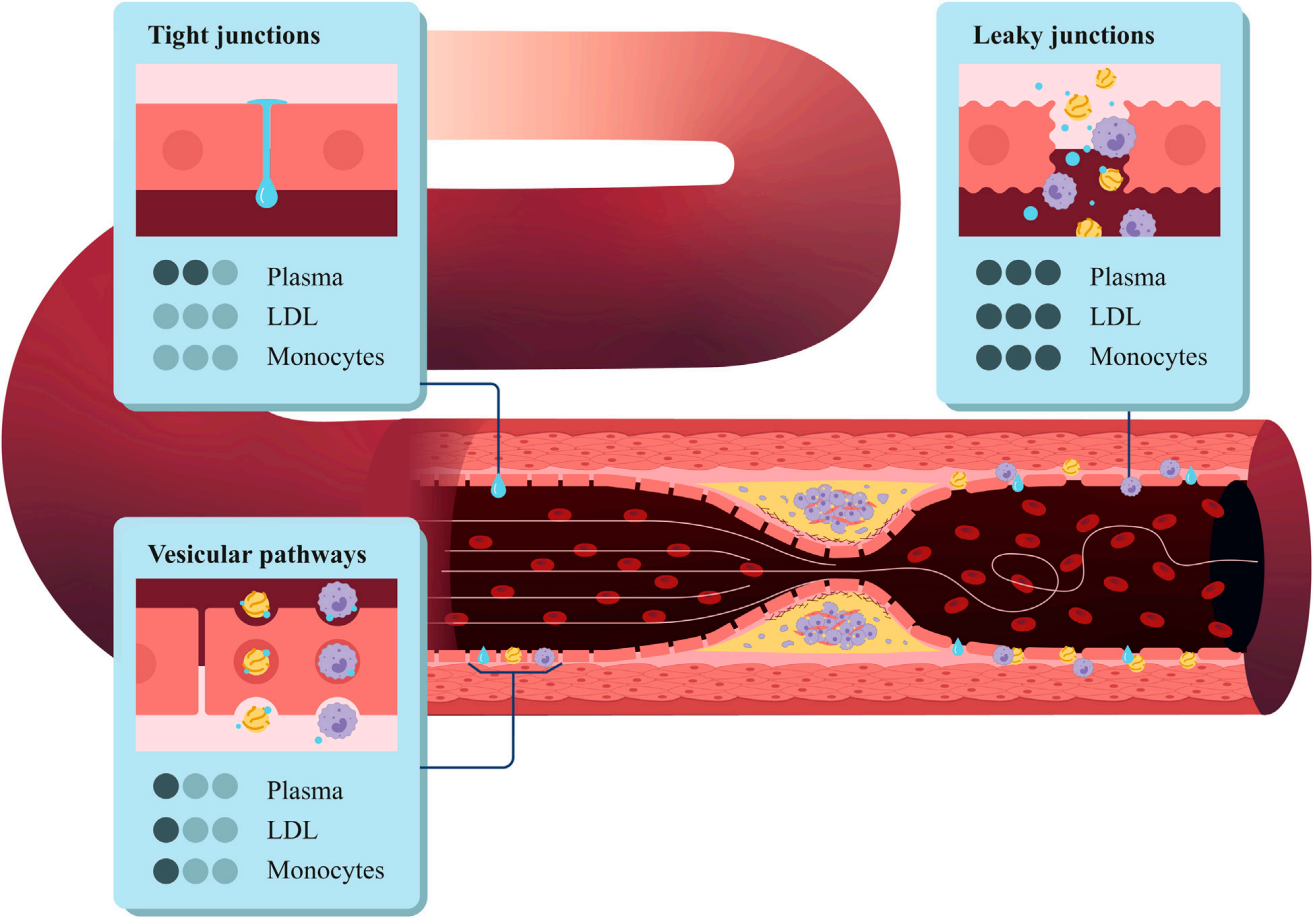
Structure of the coronary artery wall, highlighting the three types of pores in the endothelium: vesicular pathways, tight junctions, and leaky junctions.

We determined the plasma filtration flow across the endothelium based on the three-pore theory (Michel and Curry, 1999). This theory considers different pathways for substances such as plasma or LDL to cross the endothelium: normal junctions (nj) , leaky junctions (lj) , and vesicular pathways (vp) (see Figure 2). Normal junctions are the tight spaces between healthy endothelial cells and usually have an approximate size of 2−4 nm . However, in areas with oscillatory or disturbed blood flow, the mechano-sensing of endothelial cells is altered. This leads to a change in their shape, making them more circular and forming leaky junctions, which are believed to have an approximate size of 20 nm .

Then, we can apply the three-pore model to Equation 4 and compute the total flow across the endothelium as the sum of the flow across each pore type (Equation 5):

$$J_{v}=J_{v,nj}+J_{v,lj}+J_{v,vp}$$ (5)

We considered the plasma flow across the vesicular pathways to be negligible (Olgac et al., 2008), so the equation to compute the plasma flow across the endothelium can be expressed as follows (Equation 6):

$$J_{v}=J_{v,nj}+J_{v,lj}=L_{p,nj}\Delta P_{\mathrm{end}}+L_{p,lj}\Delta P_{\mathrm{end}}$$ (6)

where $L_{p,nj}$ and $L_{p,lj}$ represent the hydraulic conductivities of the membrane attributed to normal junctions and leaky junctions, respectively. Pressure drop at the endothelium was obtained from the experimental study conducted by Tedgui and Lever (1984), which analyzed the pressure drop in vessels with and without endothelium. The study observed that the presence of endothelium resulted in a pressure drop of 21 mmHg . For a more detailed explanation of how these variables are calculated, refer to Supplementray Appendix SA1.

#### 2.3.2 LDL filtration

The flux of LDL in the wall $\left(N_{\mathrm{LDL}}\right)$ was computed through Equation 7:

$$N_{\mathrm{LDL}}=-D_{\mathrm{LDL}}\nabla C_{\mathrm{LDL}}+u_{p}C_{\mathrm{LDL}}$$ (7)

In this equation, the first term represents the diffusion of LDL, and the second term accounts for LDL convection. Here, $D_{\mathrm{LDL}}$ is the diffusion coefficient, and $C_{\mathrm{LDL}}$ is the LDL concentration. The temporal evolution of LDL concentration in the artery wall was modeled using the convection-diffusion-reaction equations (Equation 8):

$$\frac{\partial C_{\mathrm{LDL}}}{\partial t}+\nabla \cdot \left(-D_{\mathrm{LDL}}\nabla C_{\mathrm{LDL}}\right)+k_{\text{lag},LDL}\cdot u_{p}\cdot \nabla C_{\mathrm{LDL}}=f_{C_{\mathrm{LDL}}}\left(\cdots \;,C_{\mathrm{LDL}},\cdots \;\right)$$ (8)

where $k_{\mathrm{lag,LDL}}$ is a coefficient that quantifies the solute lag in the arterial wall. The first term in Equation 8 corresponds to the temporal variation of LDL concentration in the arterial wall, while the second and third terms represent the diffusion and convection of LDL, respectively. Lastly, the reactive term accounts for the oxidation of LDL within the wall. $C_{\mathrm{LDL}}$ is the concentration of LDL and $D_{\mathrm{LDL}}$ its diffusion coefficient in the arterial wall, assumed as isotropic and with a value of $1e-9{\text{m}}^{2}/\text{s}$ (Table 3). The convection of LDL in a free medium is not the same as in a porous one like the arterial wall. Thus, $k_{\text{lag},LDL}$ limits the convection in the arterial wall related to the convection in a free medium, and it is named the solute lag coefficient of the considered substance in the arterial wall (Dabagh et al., 2009; Olgac et al., 2008; Sun et al., 2006).

As boundary conditions for the filtration process of the LDL we defined a specific flux at the endothelium based on the three-pore theory combined with the Kedem-Katchalsky equations (Kedem and Katchalsky, 1958), and a determined LDL concentration at the EEL.

To compute the LDL flux across the endothelium, we used an expression proposed by Kedem and Katchalsky (1958), which highlights that the total solute flux is the combination of a diffusive and a convective flow (Equation 9):

$$J_{s}=\underset{\begin{array}{c} \text{diffusive}\\ \text{term} \end{array}}{\underbrace{P_{L}\Delta C}}+\underset{\begin{array}{c} \text{convective}\\ \text{term} \end{array}}{\underbrace{J_{v}\left(1-\sigma_{f}\right)\bar{C}}}$$ (9)

where $P_{L}$ is the permeability of the endothelium, ΔC is the difference of concentration between the lumen and intima, $\sigma_{f}$ a membrane reflection coefficient $\left(\sigma_{f}\right)$ , representing the membrane’s resistance to solute passage, and $\bar{C}$ is the mean of the concentration between lumen and intima. We used a variant of Equation 9 that has also been used by other authors (Patlak et al., 1963; Tarbell, 2003), expressing Equation 9 in terms of the Peclet number (Equation 10):

$$J_{s}=P_{L}\Delta C\left\{\frac{Pe}{e^{Pe}-1}\right\}+J_{v}\left(1-\sigma_{f}\right)\bar{C}$$ (10)

where the Peclet number is a dimensionless number expressing the ratio of convective to diffusive transport. It is defined as (Equation 11):

$$Pe=\frac{J_{v}\left(1-\sigma_{f}\right)}{P}$$ (11)

Due to the size of LDL molecules (20 nm), the transport across normal junctions is zero. In addition, some studies have shown that the transport of LDL across the vesicular pathways is approximately 10% of the transport across the leaky junctions (Cancel et al., 2007) (Equations 12–14).

$$J_{s}=J_{s,nj}+J_{s,lj}+J_{s,vp}$$ (12)

$$J_{s}=1.1J_{s,lj}$$ (13)

$$J_{s,lj}=P_{L,lj}\Delta C\left\{\frac{Pe_{lj}}{e^{Pe_{lj}}-1}\right\}+J_{v,lj}\left(1-\sigma_{f,lj}\right)\bar{C}$$ (14)

Further explanation for the computation of the flix of LDL can be seen in Supplementray Appendix SA1.

Taking all this into account, we computed the oxLDL concentration solving in 2D Equations 7, 8 for the arterial wall using a time-dependent simulation.

### 2.4 Agent-based model (ABM)

**FIGURE 3:**
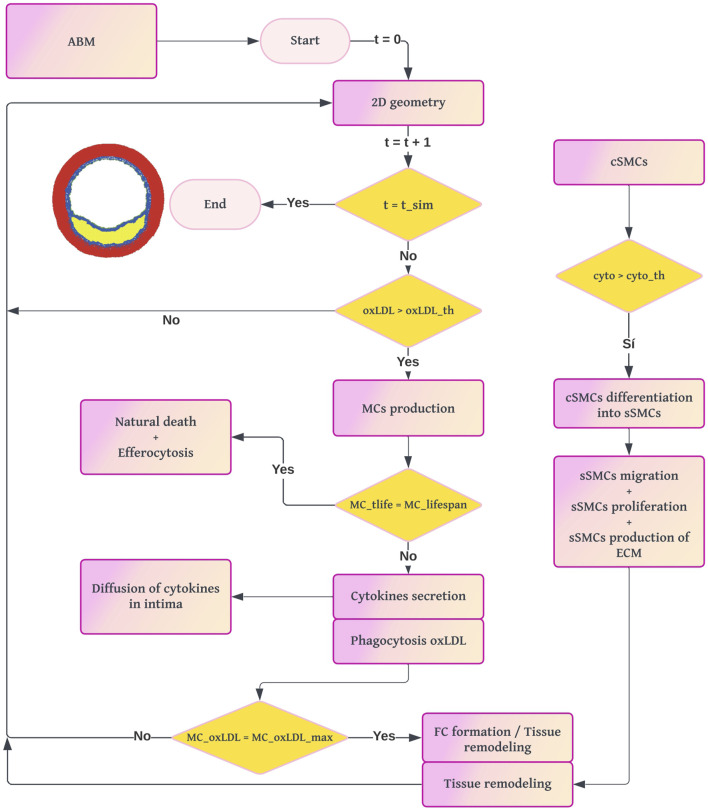
Workflow diagram of the ABM. The process begins with the 2D cross-section from the mass transport model. For each loop until the simulation time of the ABM is reached, if there is a pathological concentration of oxLDL, the model computes the probability of production of MCs and their dynamics. The formation of FCs leads to the appearance of a necrotic-lipidic core, and the secretion of cytokines triggers the vSMC activity.

The ABM was developed in NetLogo 6.1 (Wilensky, 2019) to simulate the cellular processes involved in atherosclerosis and to compute the growth of the arterial wall. The workflow of the ABM (Figure 3) begins by importing the geometry and oxLDL concentration from the 2D mass transport model (Figure 1). The domain consists of a regular grid measuring 130×130 μm , discretized into square cells of 20μm .

The cellular agents considered are ECs, MCs, FCs, and vSMCs. Both agents and lattice sites have predefined settings and behaviors depending on their type. For example, MCs can phagocytize and move, whereas FCs cannot. From now on, we will refer to the lattice sites as patches, following Netlogo’s nomenclature. Additionally, we will distinguish between simulated time (the time representing pathology in our model) and real simulation time (the actual computation time required by our machine).

The amount of oxLDL in the arterial wall is crucial for sustaining the immune response, so oxLDL is updated at each coupling interval. The models are coupled every 2 years of simulated time to balance accuracy and computational cost. After each 2-year interval, the current geometry is segmented and used to reconstruct the 3D geometry.

The ABM operates in a temporal loop where each iteration represents 1 day of simulated time. At the end of each day, the probabilities of various cellular events and the concentrations of all substances are recalculated. This process repeats until the total simulated time reaches 10 years, which is sufficient to observe both tissue growth and remodeling.

Regarding computational efficiency, simulating 10 years of pathology required 135 h of real simulation time on an Intel(R) Core(TM) i7-10700K CPU @ 3.80 GHz.

Due to the stochastic nature of most expressions describing the biological processes in the ABM (e.g., probabilities of SMC proliferation and migration), all output values were averaged over multiple simulations. The number of simulations (N) was selected to balance variability among ABM runs and computational cost. Specifically, 10 simulations were performed to minimize the standard deviation while efficiently managing computational resources.

#### 2.4.1 Initialization of the ABM

The geometric features of the ABM were derived from the 2D geometry of the mass transport model, representing a healthy cross-section of the coronary artery. In the mass transport model membranes like endothelium, IEL, and EEL were represented by lines, whereas in the ABM they were considered monolayers due to the nature of a discrete model. Thus, the first row of cells in the lumen adjacent to the intima was designated as the endothelium, and the first row of vSMCs in the media layer adjacent to the intima was designated as the IEL. The oxLDL concentration, imported from the mass transport model, remains constant until the next coupling event. During this interval, it does not undergo diffusion or elimination from the system. To align the continuous and discrete models, a mesh-matching process was applied, averaging patch values where multiple values from the continuous model overlapped.

Based on the resolution limit of the OCT imaging technique (10–20 μ m) (Prati et al., 2010), the simulation domain was divided into square cells measuring 20×20 μ m. Each cell can contain either one EC, FC, or vSMC. Additionally, each cell can simultaneously host 1 MC, ECM, and soluble substances such as oxLDL and cytokines. MCs are allowed to share the space with other cell types due to their crucial role in tissue remodeling. Under pathological conditions, these MCs migrate to the damaged area and differentiate into FCs, driving tissue remodeling.

Therefore, we determined the membrane (endothelium, IEL, EEL) or layer (intima or media) to which each set of patches belongs. Patches belonging to the endothelium were seeded with ECs. Patches in the intima layer initially contained no cells, only oxLDL and ECM concentrations. The media layer was filled with vSMCs in their contractile phenotype (cSMCs).

For substances not imported from the mass transport model, such as collagen density and cytokine concentration, initial values were set based on literature (Hernández-López et al., 2021) ( $ECM^{t=0}=2.35\times 10^{-5}{\text{ kg/m}}^{3}$ and $0{\text{ mol/m}}^{3}$ , respectively), reflecting a healthy baseline where pro-inflammatory cytokines are not yet present. The initial values of the parameters to initialize the model are summarized in Table 4. Cytokines diffusion on the ABM was modeled using a finite difference discretization of the diffusion equation in two dimensions, using an explicit method for approximating the partial differential equation (Equation 15).

$$C_{\text{cyto},i,j}^{t+\Delta t}=C_{\text{cyto},i,j}^{t}+D\Delta t\left(\frac{C_{\text{cyto},i+1,j}^{t}-2C_{\text{cyto},i,j}^{t}+C_{\text{cyto},i-1,j}^{t}}{\Delta x^{2}}+\frac{C_{\text{cyto},i,j+1}^{t}-2C_{\text{cyto},i,j}^{t}+C_{\text{cyto},i,j-1}^{t}}{\Delta y^{2}}\right)$$ (15)

**TABLE 4:** Initial ABM parameters.

Parameter	Value
Patch size (μm×μm)	20×20
Step time (day)	1
Inner radius (μm)	1,800
Outer radius (μm)	2,500
Monocyte concentration in blood $\left(\text{mol}/{\text{m}}^{3}\right)$	$550\times 10^{9}$
Initial ECM density $\left({\text{kg/m}}^{3}\right)$	$2.35\times 10^{-5}$
Diffusion coefficient of cytokines	0.3
Diameter of macrophages (μm)	10
Diameter of foam cells (μm)	20

#### 2.4.2 Cellular processes and behavioral rules

The modeled cellular events included the recruitment of monocytes and their differentiation into MCs, the phagocytosis of oxLDL by MCs, the secretion of cytokines by MCs, the differentiation of MCs into FCs and their subsequent death, the activation and phenotype change of vSMCs, their migration, proliferation and apoptosis, and the generation and degradation of ECM. Each cellular event was governed by specific rules, which were categorized into deterministic and stochastic.

Deterministic rules calculate the amount of a specific substance at a given patch and time point, based on generation or degradation ratios. Stochastic rules, on the other hand, determine the probability of a cellular event occurring. The different probabilities were calculated using sigmoid functions, which outputted a value between 0 and 1 (Allen, 2010; Banks, 2013; Albano et al., 2022). These rules were often modulated by ratios derived from experimental studies. For instance, if the differentiation rate of cSMCs into synthetic sSMCs under atheroprone conditions was 0.4, the sigmoid function was adjusted to reflect a maximum probability of 0.4, incorporating the differentiation ratio in the numerator. In the sigmoid function, the slope (k) was calibrated using data from experimental studies and our own experimental experience. Variable C represents the concentration of a substance, depending on the specific cellular event being modeled.Table 5

**TABLE 5:** Global ABM parameters.

Parameter	Description	Value	References
$r_{\mathrm{diff}}$ (1/d)	Monocytes differentiation rate	1	Cilla et al. (2012)
$r_{\mathrm{prolif}}$ (1/d)	Proliferation rate	0.24	Boyle et al. (2011)
$r_{\mathrm{apop}}$ (1/d)	Apoptosis rate	$1.9008\times 10^{-5}$	Escuer et al. (2019)
$r_{gen_{\mathrm{ECM}}}$ (kg/d)	ECM generation rate	$2.1384\times 10^{-16}$	Zahedmanesh et al. (2014)
$r_{deg_{\mathrm{ECM}}}$ (1/d)	ECM degradation rate	1/30	-
$C_{mono\text{\_}blood}$ $\left(\text{mol}/{\text{m}}^{3}\right)$	Monocytes in blood	$550\times 10^{9}$	Khan and Khan (2009)
$m_{r}$ $\left({\text{m}}^{4}/\left(\text{oxLDL}\cdot \text{d}\right)\right)$	Monocyte recruitment rate	$6.636\times 10^{-4}$	Steinberg et al. (1997)
$m_{d}$ (1/d)	Monocyte differentiation rate	$9.94\times 10^{-2}$	Bulelzai and Dubbeldam (2012); Hernández-López et al. (2021)
$r_{secr_{\mathrm{cyto}}}$ $\left(\text{cyto}\cdot {\text{m}}^{3}/\left(\text{MC}\cdot \text{cyto}\cdot \text{d}\right)\right)$	Secretion rate of cytokines	$2.592\times 10^{-5}$	Hernández-López et al. (2021)
$r_{deg_{\mathrm{cyto}}}$ (1/d)	Degradation rate of cytokines	2	Zhao et al. (2005); Hernández-López et al. (2021)
$r_{secr_{\mathrm{MMPs}}}$	Secretion rate of MMPs	$2.592\times 10^{-6}$	-
$r_{deg_{\mathrm{MMPs}}}$	Degradation rate of MMPs	2	-
$oxLDL_{MC_{\max}}$	Maximum oxLDL for macrophages	$6.7\times 10^{-15}$	-
$r_{\mathrm{phago}}$	Phagocytosis rate	$2.1168\times 10^{-18}$	-

##### 2.4.2.1 Monocyte recruitment and differentiation into macrophages

Monocytes are recruited to areas with high oxLDL concentrations and differentiate into MCs as an immune response. This process was modeled using the probability of an MC appearance in the pathological area, represented by a sigmoid function (Equation 16).

$$p_{{produce}_{MC}}=\frac{m_{d}}{1+e^{-k\cdot \left(C_{\mathrm{oxLDL}}-C_{oxLDL_{0}}\right)}}$$ (16)

Here, $m_{d}$ is the differentiation ratio from monocytes to macrophages, k is the slope of the transition in the sigmoid function, computed using the recruitment ratio of monocytes $\left(m_{r}\right)$ and the concentration of monocytes in the blood $\left(C_{\mathrm{mono-blood}}\right)$ . $C_{\mathrm{oxLDL}}$ is the concentration of oxLDL, and $C_{oxLDL_{0}}$ is the reference concentration.

##### 2.4.2.2 Macrophages’ activity

Macrophages updated their lifetime 1 day every step-time. While they were alive, they engulfed oxLDL and produced cytokines according to deterministic rules (Equation 17) based on a phagocytosis ratio $\left(r_{\mathrm{phago}}\right)$ and a secretion ratio $\left(r_{secr_{\mathrm{cyto}}}\right)$ respectively.

$$\text{if }t_{\text{life}}<t_{\text{lifespan}}\;\left\{\begin{array}{c} {\text{oxLDL}}_{MC}^{t}={\text{oxLDL}}_{MC}^{\left(t-1\right)}+r_{\mathrm{phago}}\cdot {\text{oxLDL}}_{\mathrm{patch}}^{\left(t-1\right)}\;\\ {\text{cyto}}^{t}={\text{cyto}}^{\left(t-1\right)}+r_{secr_{\mathrm{cyto}}}\cdot {\text{oxLDL}}_{\mathrm{patch}}^{\left(t-1\right)}\; \end{array}\right.$$ (17)

where ${\text{oxLDL}}_{\mathrm{patch}}^{\left(t-1\right)}$ is the oxLDL concentration at a specific cell site in the previous step time.

In addition, MCs eventually died through two possible mechanisms. The first mechanism was natural death, which occurred when an MC reached its lifespan of 100 days (Equation 18). The second mechanism occurred when MCs reached the maximum amount of oxLDL they could handle, becoming foam cells, losing their cellular functions, and releasing metalloproteases (MMPs) to the environment (Equation 19).

$$\text{if }t_{\text{life}}>t_{\text{lifespan}}\;\left\{\begin{array}{c} \text{die}\; \end{array}\right.$$ (18)

$$\text{if }\;{oxLDL}_{MC}>{oxLDL}_{MC_{\text{max}}},\;\left\{\begin{array}{c} \text{release MMPs}\;\\ \text{switch to FCs}\; \end{array}\right.$$ (19)

Cytokines produced by MCs were able to diffuse according to a chemical gradient, so this discrete diffusion of cytokines was implemented in the ABM.

##### 2.4.2.3 Smooth muscle cell behavior

cSMCs were initially located only in the media layer. These cells were in a senescent phenotype, inactive, and out of the cell cycle, with their main function being to contract and stretch, providing tone to the vessel and assisting in vasoconstriction and vasodilation. However, in regions with a pathological concentration of pro-inflammatory cytokines, cSMCs could become activated and re-enter the cell cycle. This activation enabled them to proliferate, migrate, and generate extracellular matrix by transitioning to a synthetic phenotype.

To model the probability of phenotype switching $\left(p_{\mathrm{diff}}\right)$ , we developed a rule based on the differentiation ratio from cSMCs to sSMCs $\left(r_{\mathrm{diff}}\right)$ , the concentration of cytokines $\left(C_{\mathrm{cyto}}\right)$ , and the distance to the target cell site (*distance*) and a differentiation ratio (Equation 20).

$$p_{\mathrm{diff}}=\frac{r_{\mathrm{diff}}}{1+e^{\left(C_{\mathrm{cyto}}-C_{cyto_{0}}\right)+distance}}$$ (20)

The proliferation probability $\left(p_{\mathrm{prolif}}\right)$ was influenced by the concentration of cytokines, the distance to the damaged area, and a proliferation ratio $\left(r_{\mathrm{prolif}}\right)$ derived from experimental studies (Equation 21).

$$p_{\mathrm{prolif}}=\frac{r_{\mathrm{prolif}}}{1+e^{\left(C_{\mathrm{cyto}}-C_{cyto_{0}}\right)+distance}}$$ (21)

Similarly, the rule to compute the probability of migration $\left(p_{\mathrm{migrate}}\right)$ was defined as follows (Equation 22):

$$p_{\mathrm{migrate}}=\frac{1}{1+e^{\left(Ccyto-C_{cyto_{0}}\right)+distance}}$$ (22)

Since sSMCs were now in the cell cycle, they could eventually undergo apoptosis. The rule for this cellular event was uniquely based on an experimental ratio $\left(r_{\mathrm{apop}}\right)$ (Equation 23).

$$p_{\mathrm{apop}}=r_{\mathrm{apop}}$$ (23)

Lastly, the generation of ECM was based on a production ratio $\left(r_{gen_{\mathrm{ECM}}}\right)$ (Equation 24).

$${\text{ECM}}^{t}={\text{ECM}}^{t-1}+r_{gen_{\mathrm{ECM}}}$$ (24)

Following the principle of minimum energy (Garbey et al., 2015), both migration and tissue remodeling when new cells were born, were based on an algorithm to find the shortest path to an objective. The shortest path algorithm used in our model was a modified version of the A* algorithm (Hart et al., 1968), designed to avoid obstacles in the path.

#### 2.4.3 A* algorithm

The A* algorithm is a pathfinding and graph transversal algorithm widely used to find the shortest path between nodes in a graph (Hart et al., 1968). In our model, A* was adapted to facilitate the migration of sSMCs and tissue remodeling. The key features of the A* algorithm in our context are explained below.

##### 2.4.3.1 Heuristic function

A* uses a heuristic function to estimate the cost from a given site to the target site. In our model, we used this within the sSMC migration rule and the remodeling module, where the heuristic function was designed to minimize the energy required for a cell to migrate avoiding obstacles in the path or to find an empty site that met specific characteristics.

##### 2.4.3.2 Cost function

The cost function in our adaptation of A* includes several factors.

• Cytokine concentration $\left(C_{cyto}\right)$ : The concentration of cytokines influenced cell behavior. Higher concentrations promoted migration and proliferation, thus reducing the perceived ”cost” of moving through areas with high cytokine levels, simulating the effects of chemotaxis.

• ECM density: ECM density affected cell migration. Higher ECM density increased resistance to movement, making migration more challenging in areas with denser ECM.

• Distance to damaged area: Cells were more likely to migrate towards the damaged area. The algorithm prioritized paths that minimize the distance to the target.

• Obstacles: The modified A* algorithm included logic to detect and avoid obstacles, ensuring that cells did not migrate through impenetrable regions, such as the lipid pool.

##### 2.4.3.3 Algorithm steps

1. Initialization: Start with an initial site (current position of the sSMC) and add it to the open list, which contains sites to be evaluated.
2. Evaluation: For each site in the open list, calculate the total cost f(n) as the sum of the cost to reach the site g(n) and the heuristic estimate to the target h(n) .

$$f\left(n\right)=g\left(n\right)+h\left(n\right)$$

3. Selection: Select the site with the lowest total cost from the open list and move it to the closed list, indicating it has been evaluated.
4. Expansion: Expand the selected site by evaluating its neighboring sites. For each neighbor:

• Calculate the tentative cost to reach the neighbor.

• If this tentative cost is lower than any previously recorded cost for this neighbor, update the cost and set the current site as its parent.

• If the neighbor is not in the open list, add it.
5. Termination: Repeat the evaluation and selection steps until the target site is reached or the open list is empty.

### 2.5 Model coupling

The simulation was run iteratively updating the model every 2 years with a total simulation time of 10 years. Each iteration involved the segmentation of the ABM outputs to identify the different layers in the arterial wall after growth, reconstruction of the 3D geometry, and re-running the CFD and mass transport simulations with the updated geometry. This process captured the continuous interaction between wall remodeling, hemodynamics, and oxLDL filtration.

## 3 Results

**FIGURE 4:**
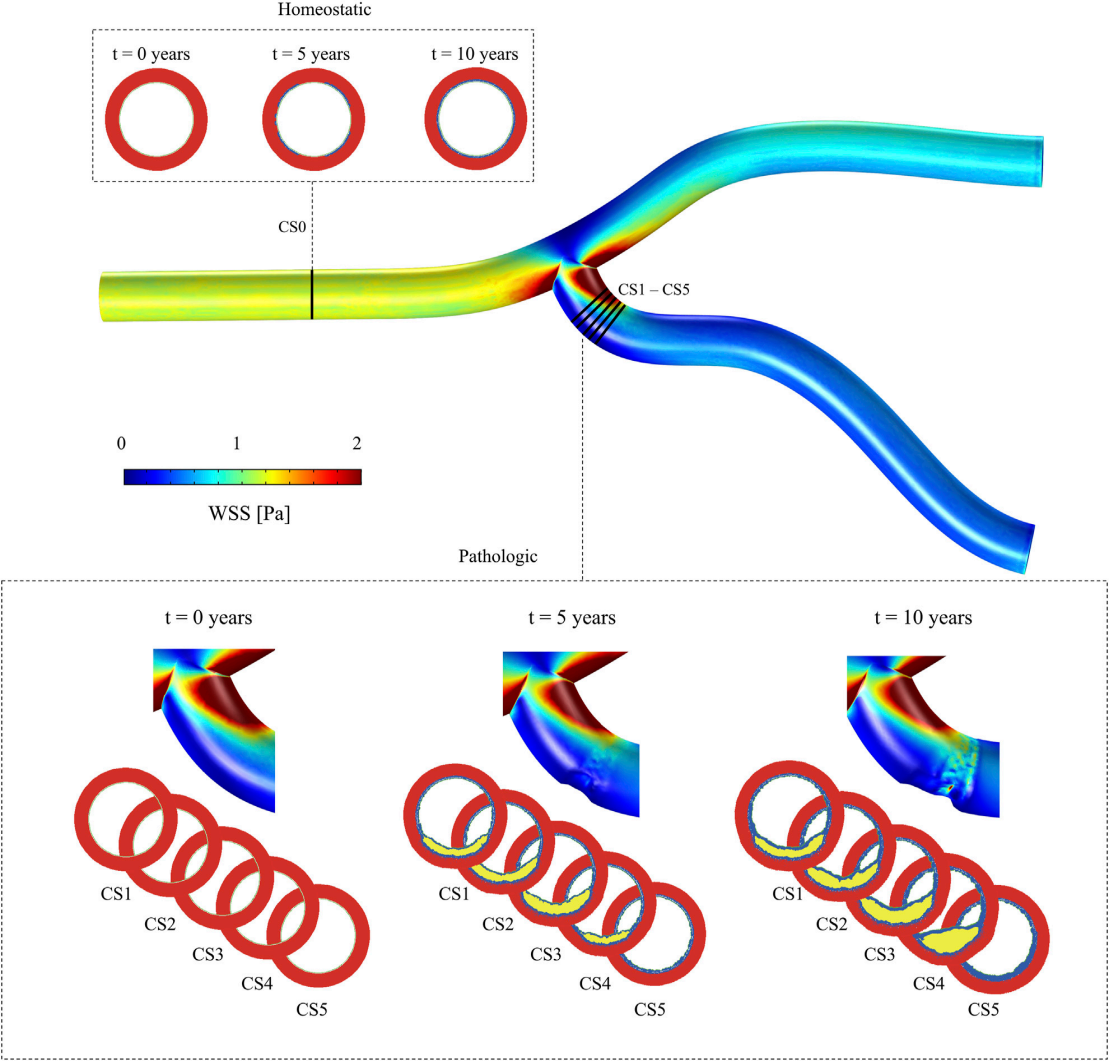
Idealized geometry of a coronary artery, illustrating the WSS distribution. Multiple cross-sectional planes are shown: CS0, located in a healthy region, to evaluate the model under homeostatic conditions, and CS1 to CS5, positioned in regions of pathological growth, to analyze behavior under disease conditions.

Given that both mechanical and chemical stimulus conditions can vary along the blood vessel, we analyzed two distinct regions (Figure 4). The first was located far from the bifurcation, an area where a parabolic flow, similar to the inlet boundary condition, was expected, representing a region less prone to atherosclerosis. To examine this region, we focused on a single cross-sectional plane (CS0). In contrast, to analyze the atherogenic region, we studied the evolution of five cross-sectional planes downstream of the bifurcation (CS1 to CS5).

### 3.1 Model response under homeostatic conditions

As CS0 is located upstream of the vessel bifurcation and due to CFD boundary conditions, the minimum WSS on this plane was 1.8Pa . Since WSS values below 1Pa are considered pathological in coronary arteries, this magnitude can be regarded as atheroprotective. Based on this WSS, the transport model produced a constant oxLDL concentration of $2.47{\text{mol/m}}^{3}$ . Under such conditions, the ABM predicted no growth, demonstrating the model’s capability to maintain homeostatic conditions (Top of Figure 4).

### 3.2 Model response under pathological conditions

**FIGURE 5:**
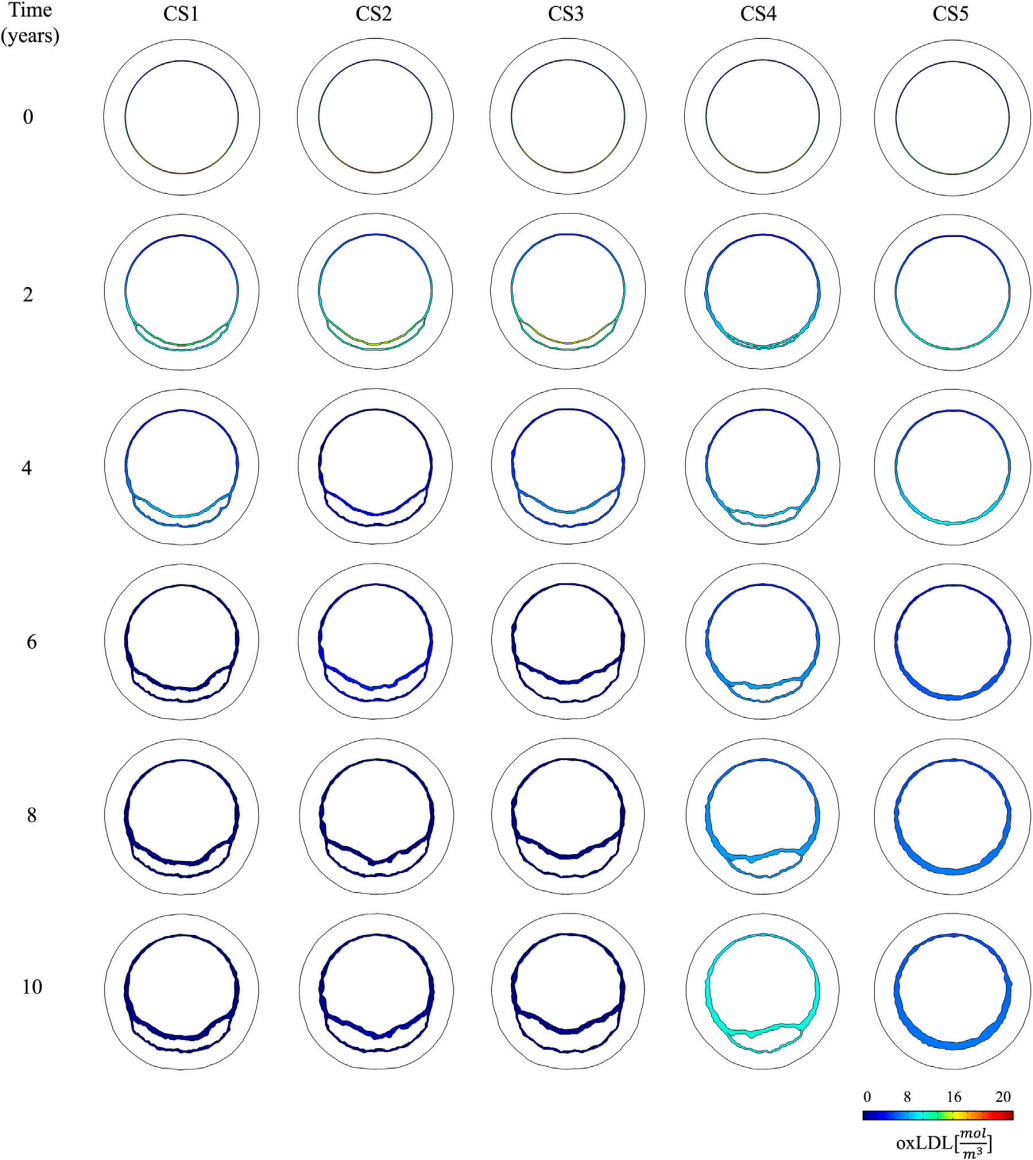
Temporal evolution of oxLDL distribution in the cross-sectional planes located in the pathological region (CS1 to CS5).

**FIGURE 6:**
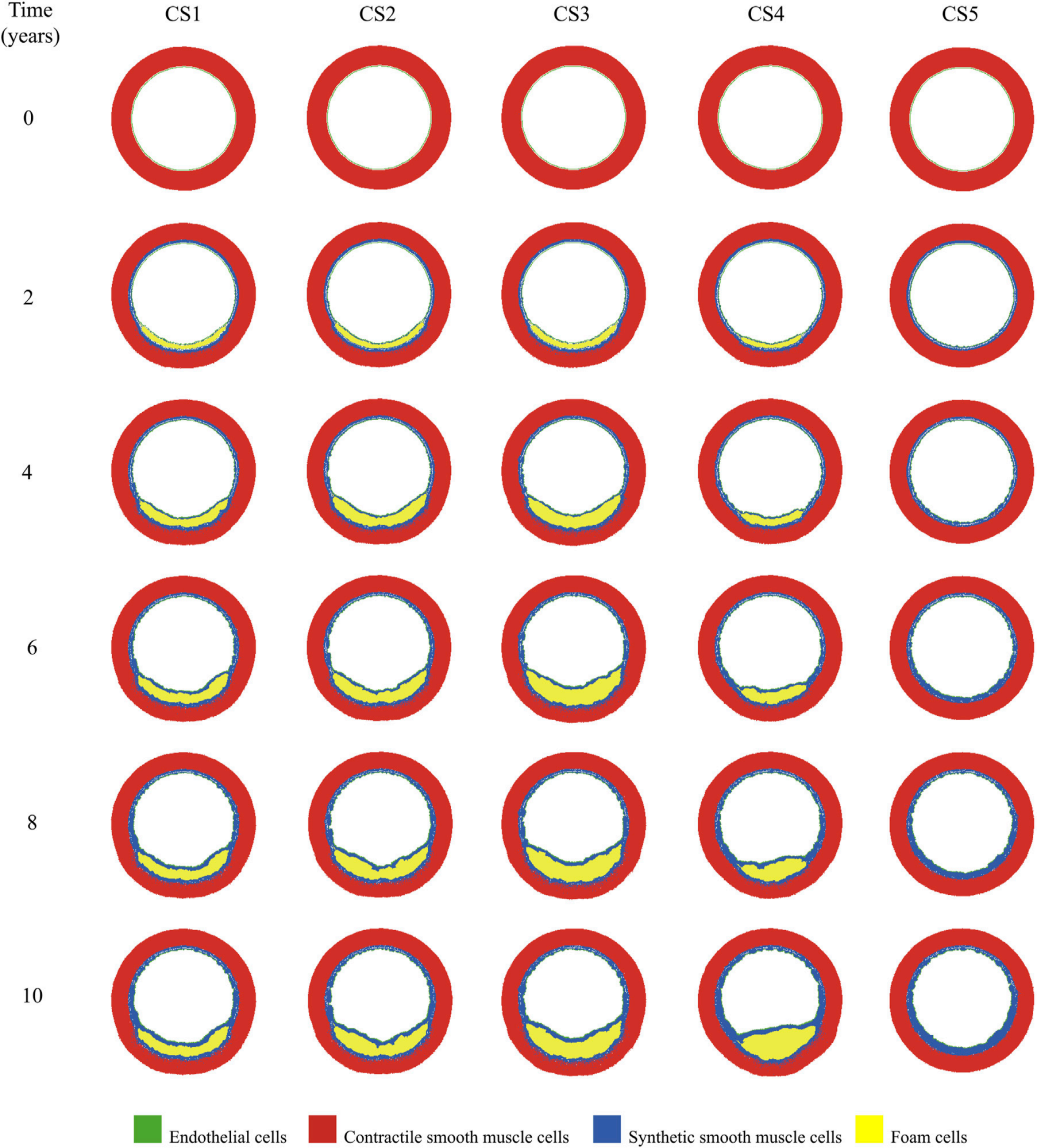
Temporal evolution of growth in the cross-sectional planes located in the pathological region (CS1 to CS5).

Cross-sectional planes CS1 to CS5, located downstream of the bifurcation, showed the lowest WSS, with minimum values of 0.0528Pa , 0.0234Pa , 0.005Pa , 0.024Pa , 0.0463Pa respectively. Regarding oxLDL concentrations, these planes showed values of $18.31{\text{mol/m}}^{3}$ , $18.45{\text{mol/m}}^{3}$ , $18.65{\text{mol/m}}^{3}$ , $17.56{\text{mol/m}}^{3}$ , $16.66{\text{mol/m}}^{3}$ respectively (Figure 5). With these atherogenic conditions, the ABM predicted different plaque growth in each CS (Bottom of Figure 4). Over time, growth in CS1, CS2, and CS3 disrupted the flow, leading to increased filtration in CS4. This disturbance caused that CS4, initially growing slower, eventually accelerated and resulted in significant plaque development (Figure 6).

**FIGURE 7:**
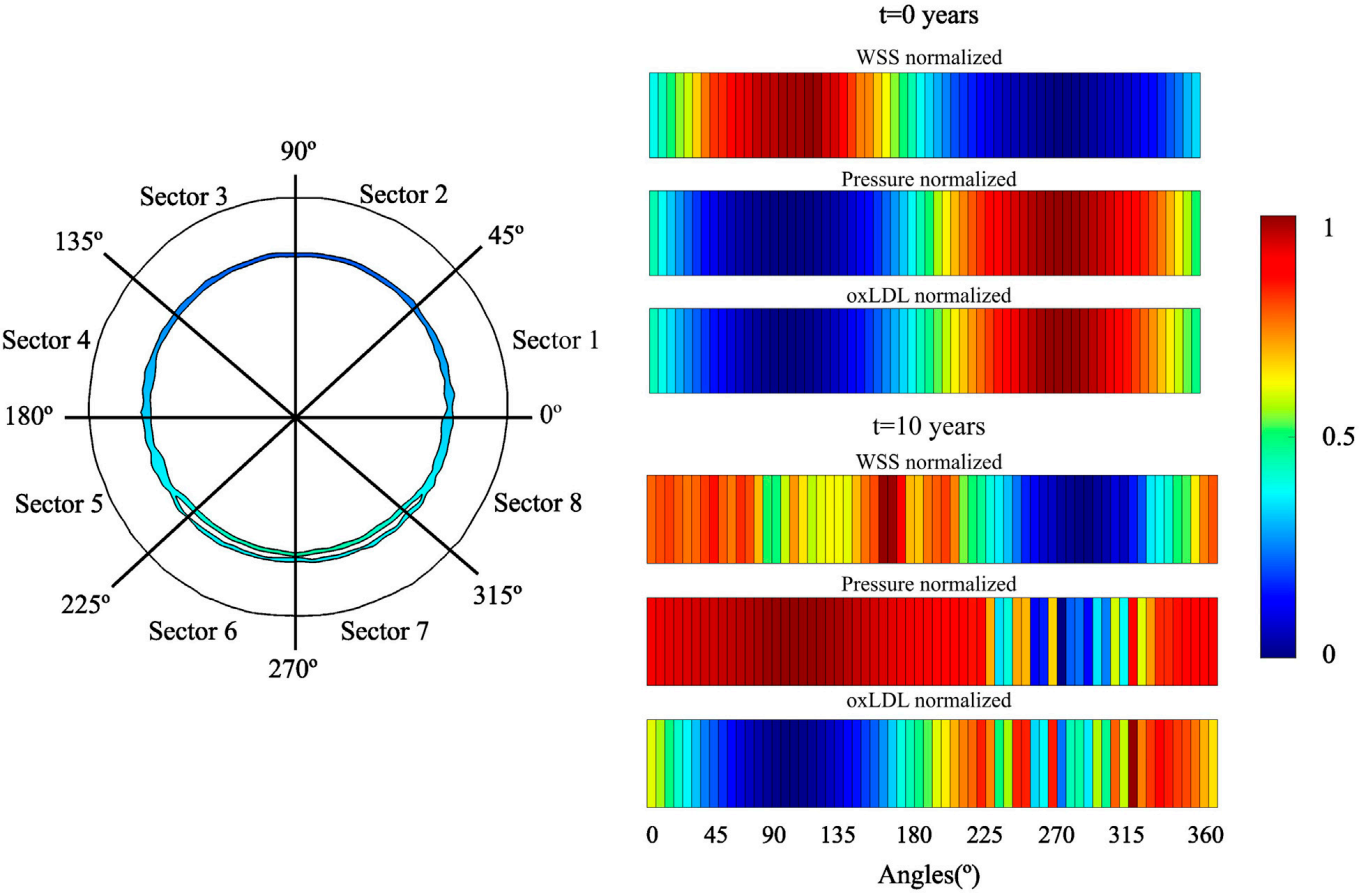
Temporal evolution of WSS, pressure and oxLDL in CS3. A two-dimensional map of distribution of these variables. For analysis, the cross-section was divided in eight sectors.

The change of phenotype of SMCs from contractile to synthetic, migration, and proliferation of sSMCs, combined with the eccentric growth of the necrotic lipid core, resulted in an outward expansion. Additionally, cellular activity in the inner region of the necrotic lipid core, considering its impermeability, led to a higher concentration of oxLDL in this area, driving a faster inward progression of the plaque. As depicted in Figure 6, CS3 was the cross-section with the highest growth, reaching a stenosis ratio (SR) of 20% after 10 years, whereas CS5 was the cross-section with the lowest SR, reaching a maximum of 8% after 10 years. Within cross-sections CS1 to CS5, a circumferential gradient of oxLDL was observed, attributed to differences in endothelial permeability driven by mechano-sensed WSS (Figure 5). Focusing on CS3, 50% of the cross-section experienced WSS below 0.5 Pa at t = 0 years (sectors 5–8) (Figure 7). This pathological scenario was further highlighted by circumferential gradients in pressure and oxLDL. Sectors with pathological WSS showed a strong correlation with higher oxLDL concentrations.

$$SR=\left(1-\frac{A_{l}}{A_{l,init}}\right)\cdot 100$$ (25)

$$LR=\left(\frac{A_{\mathrm{lipid}}}{A_{\mathrm{intima}}+A_{\mathrm{lipid}}}\right)\cdot 100$$ (26)

$$FR=\left(\frac{A_{\mathrm{fibrotic}}}{A_{\mathrm{intima}}+A_{\mathrm{lipid}}}\right)\cdot 100$$ (27)

### 3.3 Temporal evolution of the environment

**FIGURE 8:**
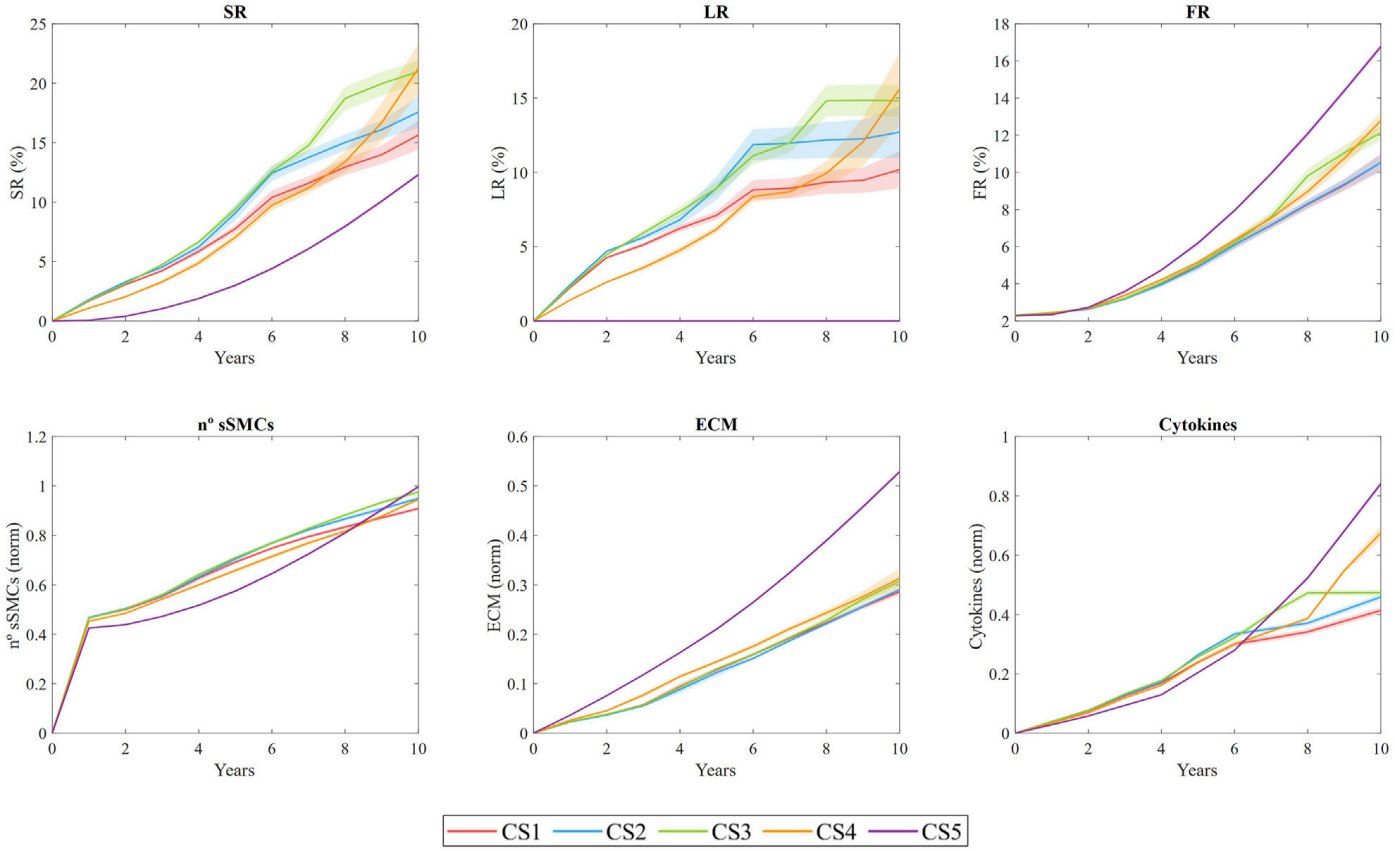
Temporal evolution of stenosis ratio (SR), lipid ratio (LR), fibrous cap ratio (FR), number of smooth muscle cells (sSMCs), extracellular matrix (ECM) density, and cytokine concentration for cross-sections CS1 to CS5. The results are obtained from N = 10 simulations, showing the mean and standard deviation.

Figure 8 shows the temporal evolution of several output variables for each cross-section. These include the stenosis ratio (SR), defined as the ratio of the current lumen area $\left(A_{l}\right)$ to the initial lumen area $\left(A_{l,init}\right)$ (Equation 25); the lipid ratio (LR), which represents the proportion of lipid area $\left(A_{\mathrm{lipid}}\right)$ relative to the total area of the intima and lipid plaque combined $\left(A_{\mathrm{intima}}+A_{\mathrm{lipid}}\right)$ (Equation 26); and the fibrous cap ratio (FR) which reflects the proportion of fibrotic tissue area $\left(A_{\mathrm{fibrotic}}\right)$ relative to the same combined area. Additionally, the total number of smooth muscle cells $\left(n_{\mathrm{sSMCs}}\right)$ (Equation 27) is given as the sum of sSMCs in each cross-section, along with the total extracellular matrix (ECM) density and the total cytokine concentration in the same cross-section.

SR shows an overall increasing trend across all sections, with a pronounced steepening of the curve in CS4. This steep increase is driven by plaque displacement resulting from the growth of sections CS1, CS2, and CS3. Specifically, CS3 underwent rapid growth during the first 6 years, primarily due to lipid-core expansion and sSMC proliferation, ultimately reaching an SR of 19% after 10 years. In CS1, CS2, and CS3, LR increased quickly during the first 6 years, with values reaching 10%, 12%, and 17%, respectively, before stabilizing, while both SR and FR continued to rise. This trend suggests a correlation between sSMC proliferation and SR, driven by cytokine-induced proliferation in the vessel. CS4 exhibited prolonged growth, with the highest rate of expansion occurring after year 8. In contrast, CS5 did not develop a necrotic core, as expected. Regarding FR, all sections showed an upward trend, with CS3 and CS5 demonstrating the most significant fibrotic tissue development relative to the intima area.

The $n_{\mathrm{sSMCs}}$ in the system increases rapidly during the first year and then continues to grow at a slower rate. It reaches its maximum value at the end of the study, suggesting that if the study had continued, the $n_{\mathrm{sSMCs}}$ would have kept increasing due to the persistent pathological stimulus, leading to a thicker fibrous cap.

ECM density also increases throughout the study, indicating that the system has not yet reached equilibrium. Since ECM is produced by sSMCs and degraded by MMPs (generated by FCs), the increasing $n_{\mathrm{sSMCs}}$ and high cytokine concentrations suggest that sSMCs continue to perceive the need to generate ECM. ECM production would likely cease once the pro-inflammatory stimuli subside.

Cytokine concentrations are closely linked to oxLDL levels, as macrophages release cytokines in response to elevated oxLDL. As the concentration of pro-inflammatory cytokines rises, sSMCs become activated and begin producing ECM. However, the increased death of MCs results in higher MMP concentrations, which degrade ECM. In regions lacking sSMCs, this degradation weakens the fibrous cap. Cross-sections CS1 to CS5 developed higher levels of pro-inflammatory cytokines than CS0, with the maximum cytokine concentration observed in the most damaged area of CS3 after 10 years.

### 3.4 Coupling CFD, mass transport and ABM

This fully coupled methodology enables the dynamic updating of WSS and oxLDL as the geometry evolves. Vessel narrowing leads to flow acceleration, which increases WSS and subsequently decreases LDL filtration into the wall. However, the emergence of a protrusion in the lumen due to growth alters the downstream flow distribution, translating the plaque downstream as WSS decreases significantly. This phenomenon is evident in CS4, which initially experienced lower oxLDL levels compared to CS3, but, as CS3 continued to grow, CS4 increased its oxLDL concentration, leading to accelerated growth and ultimately achieving SR and LR values comparable to those of CS3, demonstrating the importance of coupling these three models.

In addition, sections with faster growth during the initial years, such as CS1 and CS2, exhibited a decrease in growth rate over time. For instance, for CS1 the rate decreased from ΔSR=2% per year during the first 8 years to ΔSR=0.3% per year after year 6.

### 3.5 Sensitivity analysis

#### 3.5.1 Sensitivity to coupling time

**FIGURE 9:**
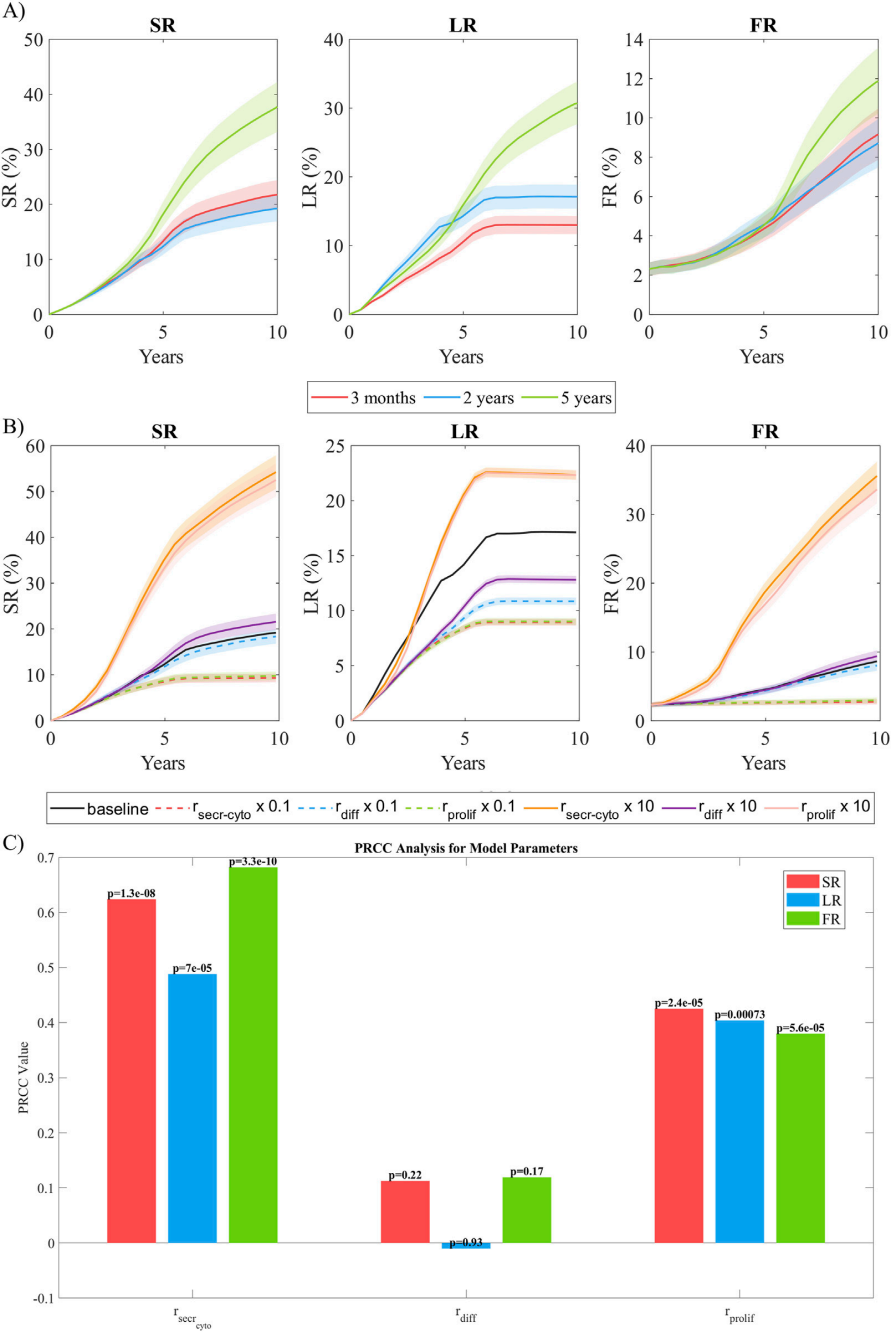
Sensitivity analysis. **(A)** Temporal evolution of SR, LR, and FR as a function of the coupling frequency between the continuous and discrete models. Coupling intervals of 3 months, 2 years, and 5 years were analyzed. **(B)** Temporal evolution of SR, LR, and FR as a function of the order of magnitude of the parameters studied ( $r_{secr_{\mathrm{cyto}}}$ , $r_{\mathrm{diff}}$ , and $r_{\mathrm{prolif}}$ ). **(C)** Partial Rank Correlation Coefficients (PRCC) of the parameters studied against the response variables SR, LR, and FR. The p-values corresponding to each correlation are displayed on the bars, indicating the statistical significance of the relationships. The results are obtained from N = 10 simulations, showing the mean and standard deviation.

In this analysis, we compared the impact of coupling time in the results. To do so, we examined three different coupling intervals in CS3: 3 months, 2 years, and 5 years. As shown in Figure 9A), both the 3-month and 2-year coupling intervals exhibited comparable performance in estimating growth. In contrast, the 5-year coupling interval predicted faster growth during the initial 5 years, followed by controlled growth in subsequent years after coupling. This behavior may be attributed to the variation of stenosis ratio (ΔSR) , where values around 5% are considered to have a significant impact on WSS. Such SR variations occur approximately every 2 years under the specific conditions of this model, making the 2-year coupling interval the optimal choice for this scenario.

#### 3.5.2 Sensitivity to parameters magnitude

To assess the model’s sensitivity to parameter variations, we selected $r_{secr_{\mathrm{cyto}}}$ , $r_{\mathrm{diff}}$ , and $r_{\mathrm{prolif}}$ due to their pivotal roles in the cellular mechanisms driving plaque growth. These parameters regulate cytokine-mediated signaling, cellular proliferation, and spatial diffusion processes, which are fundamental to atherosclerotic progression.

Figure 9B shows the temporal evolution of SR, LR and FR for every variation in the selected parameters. A reduction of cytokine secretion rate by one order of magnitude resulted in a total stenosis ratio after 10 years that was halved. This was primarily due to a 50% reduction in lipid growth and an almost complete absence of fibrous cap formation. The main reason for this behavior was the lack of cytokines in the system producing a pro-inflammatory response, leading to a significantly reduced $n_{\mathrm{sSMCs}}$ in the system. Consequently, there was insufficient ECM generation and sSMC proliferation to contribute to intimal thickening. In contrast, increasing the cytokine secretion rate by an order of magnitude resulted in a drastic increase in SR due to hyperplasia in the intima, driven by a stronger pro-inflammatory signal. Varying the differentiation rate of cSMCs to sSMCs had minimal impact on most analyzed variables, except for the LR. This was likely because the increase in the $n_{\mathrm{sSMCs}}$ primarily arose from proliferation, as this cellular event is responsible for hyperplasia in response to pro-inflammatory stimuli. However, LR was affected because it represents the proportion of the intima occupied by lipids, so with a smaller intimal area, the same amount of lipids occupied a relatively larger proportion. Variations in the proliferation rate produced effects similar to those observed with a reduced cytokine secretion rate. This similarity is likely explained by the direct relationship between cytokines and the proliferation of sSMCs, as these cells respond to cytokine concentrations as a signal indicating the need to reinforce the tissue, thereby initiating proliferation. Table 6 summarizes the relationships between the analyzed parameters and cellular behaviors identified in this sensitivity analysis.

**TABLE 6:** Summary of the sensitivity analysis results and parameter influence on cellular behavior.

Parameter	Effect on model	Main observations
$r_{secr_{\mathrm{cyto}}}\;↓$	Decreased SR, FR	50% reduction in LR, fewer sSMCs, lack of ECM
$r_{secr_{\mathrm{cyto}}}\;↑$	Increased SR	Hyperplasia due to stronger pro-inflammatory signal
$r_{\mathrm{diff}}\;↑$ or ↓	Minimal impact	Only LR affected due to relative lipid proportion
$r_{\mathrm{prolif}}\;↓$	Decreased SR, FR	Fewer sSMCs, reduced ECM, lower SR and FR
WSS ↓	Increased oxLDL filtration	Promotes lipid accumulation and inflammation
oxLDL ↑	More cytokines	Drives macrophage recruitment and inflammation
Cytokine ↑	More sSMC proliferation	Leads to hyperplasia, increased ECM production
ECM density ↑	Restricts cell migration	Influences plaque stability and remodeling

#### 3.5.3 Multi-parametric sensitivity analysis

To assess the global influence of key model parameters— $r_{secr_{\mathrm{cyto}}}$ , $r_{\mathrm{diff}}$ , and $r_{\mathrm{prolif}}$ —a comprehensive multi-parametric analysis was performed. Latin hypercube sampling (LHS) was employed to generate a robust set of 50 random parameter combinations (N = 50), ensuring an efficient and systematic exploration of the parameter space. This sampling strategy facilitated the identification of both individual and interactive effects of the selected parameters, laying the foundation for the subsequent global sensitivity analysis.

To quantify the correlation between the model parameters and the target outputs (i.e., SR, LR, FR), Partial Rank Correlation Coefficients (PRCC) were computed. PRCC was chosen because it accounts for nonlinear but monotonic relationships while controlling for the influence of other parameters, providing a robust measure of sensitivity in complex biological models (Marino et al., 2008).

Figure 9C shows the PRCC analysis, revealing that $r_{secr_{\mathrm{cyto}}}$ is the most influential parameter across the three outputs (SR, LR, and FR), exhibiting high positive correlations (approximately 0.62 for SR, 0.49 for LR, and 0.68 for FR) with p−value<0.05 , which underscores its statistically significant impact on the model. In contrast, $r_{\mathrm{prolif}}$ shows moderate positive correlations (approximately 0.43 for SR, 0.40 for LR, and 0.38 for FR) with p−value<0.05 , indicating that while its effect is significant, it is less pronounced than that of $r_{secr_{\mathrm{cyto}}}$ . Notably, $r_{\mathrm{diff}}$ exhibits only a marginal correlation (PRCC = 0.11–0.12 for SR and FR, and nearly 0 for LR with PRCC = −0.01), with p-values (e.g., 0.2194 for SR and 0.9288 for LR) that are not statistically significant. These findings suggest that, when controlling for the influence of other variables, the model outputs are predominantly driven by the parameters related to secretion and proliferation, whereas the parameter related to smooth muscle cell differentiation does not exert a direct significant effect.

### 3.6 Model validation

This methodology, integrating CFD, mass transport, and ABM to simulate plaque growth in coronary arteries under atherosclerosis, was validated against an experimental study involving individuals at high risk for atherosclerosis (Insull Jr 2009). The study focused on the population of New Orleans, a region with one of the highest cardiovascular disease (CVD) risk rates worldwide. Plaque progression data across different age groups were averaged to represent mean progression over time, allowing direct comparison with our model’s predictions. To be able to compare our model prediction against the results from the experimental study, we performed the computation until 30 years with the same coupling time.

**FIGURE 10:**
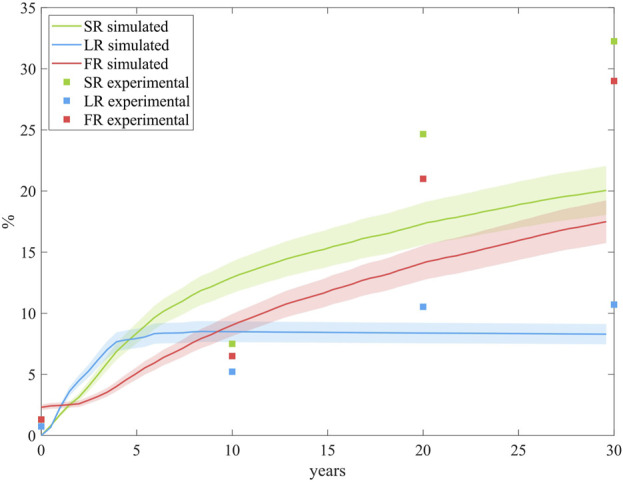
Comparison of the growth predictions from the model with the experimental data from the study conducted by Insull Jr (2009). The results are obtained from N = 10 simulations, showing the mean and standard deviation.

Figure 10 compares our predictions on SR, LR and FR with the experimental data. The results demonstrate that the model performs well, showing robust predictive capabilities. In terms of LR, the model closely matches experimental trends, achieving similar lipid-core stabilization over time. For SR and FR, the model predicts a comparable trend, accurately reflecting the proportionally lower growth of FR relative to SR. However, the model underestimates overall growth compared to the experimental study. This discrepancy may stem from differences in the pathological stimuli considered. In our model, pathological conditions are induced by performing CFD on an idealized coronary artery, with a bifurcation and initial conditions triggering a pathological scenario. In contrast, the experimental study accounts for pathological stimuli from the high-fat diet prevalent in the New Orleans population.

## 4 Discussion

The study of hemodynamics using CFD was performed to calculate the WSS. Our CFD model identified the area of lowest WSS located near the bifurcation (Figure 4). This observation is linked to the idealized geometry and symmetric boundary conditions. Despite its limitations, the purpose of this geometry was to replicate the flow conditions of a healthy coronary artery subjected to a pathological stimulus. This was achieved by simulating flow in a bifurcation, creating sufficient flow disturbances to induce a low WSS capable of damaging the endothelium, thereby allowing substances like LDL to penetrate. Furthermore, over the years simulated within the model, we observed a phenomenon commonly found in patients with atherosclerosis: the presence of small plaques often leads to additional flow disturbances, promoting the growth of new plaques downstream. This behavior is particularly evident in the temporal evolution of CS4. Although the reasons for plaque growth near bifurcations in patient-specific arteries may vary, such as differences in artery caliber or pressure, our model consistently predicts the area of lowest WSS in a coherent region. However, it is important to consider not only the location of the WSS region but also its magnitude. And, as can be observed in Figure 4, the areas of lowest WSS show values much lower than 1 Pa, which implies a severe risk factor for plaque development.

Transport phenomena are strongly linked to endothelial damage. Disturbances in WSS alter the shape of endothelial cells, increasing their permeability and allowing both plasma and LDL molecules to infiltrate the vessel wall. The oxLDL concentration obtained under homeostatic conditions in our model (2.47 ${\text{mol/m}}^{3}$ ) aligns closely with the values reported in the study by Hernández-López (2023). This agreement suggests that the model accurately replicates physiological transport processes and lipid dynamics within coronary arteries under non-pathological conditions. The presence of sSMCs observed after 10 years of simulation (Figure 4) is attributed to the pathological threshold for oxLDL being set at 0. As a result, any presence of oxLDL in the system triggers an immune response. However, unless the oxLDL concentration reaches sufficiently high levels for a sustained period, this immune response does not lead to significant growth or the formation of a lipid core.

As illustrated in Figure 7, regions with lower WSS exhibit higher pressure, indicating a reduced pressure drop across the endothelium. Consequently, increased permeability correlates with greater pressure drops. These damaged regions also provide easier pathways for LDL molecules to transmigrate across the endothelium, leading to localized areas with elevated concentrations of oxLDL within the vessel wall.

None of the figures display oxLDL concentrations in the media layer. This choice was made to enhance the clarity of the figures, given the small thickness of the intima. However, it is important to emphasize the sharp decrease in oxLDL concentration from the intima to the media layer, which underscores the significant barrier posed by the IEL to oxLDL molecule transport. These findings are consistent with the fact that the intima layer is more permeable than the IEL, causing LDL molecules to primarily move circumferentially rather than radially once they reach the IEL.

With the proposed rules about MCs’ dynamics, the model successfully mimicked the process of oxLDL phagocytosis in the damaged area, leading to the formation of FCs. In contrast, in healthy areas, MCs underwent natural cell death, preventing the formation of FCs. During the phagocytosis process, MCs secreted pro-inflammatory cytokines, which play a crucial role in the activation of sSMCs, as well as in their migration and proliferation.

Once these cells switch phenotype to sSMCs, they migrate, proliferate, and produce ECM. However, as the lipid core grows due to the formation of FCs, MMPs are released, leading to ECM degradation. The transformation of MCs into FCs and the presence of MMPs prevent the formation of a significant fibrous cap until the growth of the lipid core stabilizes. Across all the cross-sections analyzed, once the lipid core stabilizes, the fibrous cap begins to thicken. This phenomenon also occurred in the model developed by Corti et al. (2020).

During their migration process, sSMCs utilized a shortest path-finding algorithm to identify and move toward the damaged patch in need of repair. The adaptation of the A* algorithm to successfully find the shortest path allowed the effective implementation of the minimum-energy principle, intrinsically present in cell remodeling (Garbey et al., 2015). According to the minimum-energy principle, the immune response exerted by sSMCs should be influenced by distance, as closer cells are more likely to sense the damage signals from the damaged area. Consequently, incorporating distance into the computation of the probabilities for different cellular events is crucial for accurately simulating this response. For instance, in the proliferation process, the proximity to the damaged area plays a critical role, with cells nearer to the damage exhibiting a higher probability of hyperplasia. This dynamic underscores the significant impact of localized damage on promoting cellular proliferation in the surrounding tissue, contributing to the overall progression of the wall growth.

The remodeling algorithm revealed a natural and organic appearance of Glagov’s remodeling (Glagov et al., 1987). To the best of our understanding, this is the first *in silico* framework where Glagov’s remodeling is observed without being hard-coded.

According to the temporal evolution of SR, the $n_{\mathrm{sSMCs}}$ , and the total concentration of cytokines, we observed that the increase in SR within the model is primarily driven by the proliferation of sSMCs, which is stimulated by cytokine concentration. Additionally, the development of the lipid core plays a significant role in SR progression.

The collagen increment was most pronounced in the areas immediately surrounding the necrotic core and the fibrous cap. This localized increase in collagen concentration contributed to the mechanical stability of the plaque, reducing the likelihood of rupture in regions with higher collagen deposition.

As a consequence of the stochastic rules developed in this model, it predicts the formation of an irregular fibrous cap, with significant variability in its thickness, which aligns with clinical observations of real atheromatous plaques. This irregularity in the fibrous cap is critical, as it can influence plaque stability and the risk of rupture.

To balance accuracy and computational cost, performing a sensitivity analysis on the coupling interval is essential. The highest accuracy would be achieved by coupling the models daily, updating the geometry, WSS, and oxLDL at the end of each simulation step of the ABM. However, this approach is computationally impractical. Therefore, it is necessary to find a trade-off between accuracy and computational efficiency. To address this, we analyzed coupling intervals of 3 months, 2 years, and 5 years. The results demonstrated the robustness of the model when coupling every 2 years, achieving performance comparable to 3-month coupling while significantly reducing computational costs. We suggest that, since WSS changes with vessel growth, a coupling interval triggered by at least a ΔSR=5% is advisable. In our model, this ΔSR=5% is reached approximately every 2 years, consistent with the observations reported in Hernández-López (2023).

In a model, it is common to find that certain parameters have a greater influence on the final outcome. Since our model was designed to replicate plaque growth, parameters directly affecting growth are expected to have the highest impact. To assess this, we analyzed the model’s sensitivity to variations of one order of magnitude in the cytokine secretion rate, vSMC differentiation rate, and sSMC proliferation rate. This analysis highlighted that $r_{secr_{\mathrm{cyto}}}$ and $r_{\mathrm{prolif}}$ have comparable impacts on the model’s response, as both parameters are part of the same chain of cellular events: cSMCs sensing cytokine gradients, undergoing phenotype switching, migrating, and proliferating in response to cytokine concentrations. In contrast, $r_{\mathrm{diff}}$ had a much smaller influence on growth, as hyperplasia in our model was found to be primarily driven by the proliferation of sSMCs rather than their phenotypic transition.

Overall, this analysis highlights the critical role of cytokine dynamics and ECM-related processes in driving the observed growth patterns, emphasizing the need for accurate calibration of these parameters to achieve realistic predictions.

Nevertheless, several important limitations remain. First, while most current atherosclerosis studies utilize patient-specific geometries, our model relies on an idealized geometry, which was necessary for the initial development of the framework. It is well recognized that results derived from idealized geometries may differ when applied to realistic ones. For instance, a notable feature of our model’s predictions is that most plaques grow symmetrically, a consequence of the absence of tortuosity in the geometry used. As such, employing an idealized coronary artery geometry may limit the model’s applicability to patient-specific conditions. Future work should focus on integrating patient-specific geometries to enhance the model’s clinical relevance.

Additionally, assumptions such as steady-state flow and Newtonian fluid behavior may not fully capture the complexities of *in-vivo* blood flow. Incorporating the cardiac cycle into the CFD model and more complex rheological properties could enhance accuracy. This could be further improved by utilizing atheroprone markers such as time-averaged wall shear stress or the oscillatory shear index. Nonetheless, these simplifications are widely used by most authors in the development of atherosclerosis models.

The iterative nature of the model, particularly the coupling between the CFD and ABM components, and the computation of the short-path finding algorithm, requires significant computational resources, which may limit its accessibility for widespread use.

Currently, many researchers are investigating additional factors that may influence plaque growth apart from the magnitude of WSS. Although the most widely accepted biomechanical marker by the scientific community has been the magnitude of WSS, other markers such as the Oscillatory Shear Index (OSI), transverse WSS (transWSS), WSS direction, and gradient are being proposed. In this study, we assumed the widely accepted hypothesis that endothelial cells change shape, making the endothelial barrier more porous. This shape change follows a model based on experimental data provided by Levesque et al. (1986), considering WSS as the main driver of endothelial damage. Future work could involve adjusting this model to include other variables such as OSI, but this remains a topic for further research.

We established a constant concentration of monocytes in the lumen of $500\cdot 10^{9}{\text{mol/m}}^{3}$ , according to Khan and Khan (2009). This concentration was used to compute the probability of producing MCs in the intima. However, the concentration of monocytes in blood could be another variable worth studying. As suggested by Gu et al. (2019), hematopoiesis of immune cells in the blood is influenced by the environment, including conditions such as hypercholesterolemia. This highlights the importance of considering blood monocyte levels when evaluating the production of MCs in the intima, as the systemic environment significantly impacts immune cell generation.

This study enhances the level of detail in modeling atheroma plaque progression. A key innovation compared to other works in the field is the inclusion of greater specificity in the types of cells and cellular events modeled. This feature could provide a foundation for future research to evaluate the effectiveness of different therapies in slowing plaque progression.

Despite its limitations, the model serves as a powerful tool for predicting initiation and progression of the plaque, detecting high-risk regions and investigating potential therapeutic strategies. Future research should focus on refining the model and validating its predictions with clinical data to pave the way for its use in personalized medicine.

## 5 Conclusion

In this study, we developed a fully coupled model that serves as an innovative tool for investigating the underlying mechanisms of atherosclerosis, offering valuable insights for developing therapeutic and preventive strategies. This model simulates complex interactions between hemodynamics, mass transport, and cellular responses, providing a holistic understanding of disease progression. Although not yet validated against experimental or clinical data, it establishes a detailed framework for understanding atherosclerosis mechanisms and exploring potential therapeutic strategies. The findings serve as a foundation for future work, including model validation and refinement based on experimental data. Additionally, the integration and adaptation of the A* algorithm effectively modeled the complex migration and remodeling behavior of sSMCs in response to injury and cytokine signaling, providing a robust framework for understanding cellular dynamics in vascular pathology.

## Data Availability

The original contributions presented in the study are included in the article/Supplementary Material. Further inquiries can be directed to the corresponding author.
