# Supplementary material for: Fully coupled hybrid in-silico modeling of atherosclerosis: A multi-scale framework integrating CFD, transport phenomena and agent-based modeling
Source: Front Bioeng Biotechnol. 2025 Mar 28;13:1549104. doi: 10.3389/fbioe.2025.1549104 (PMC11986476; doi:10.3389/fbioe.2025.1549104)
Supplement: Supplementary file 1 [file Supplementaryfile1.pdf]

1 Appendix

Here we provide a detailed explanation of the mass transport model.

1.1 Mass transport model

We extracted several cross-sections along with their corresponding WSS data from the 3D idealized geometry used in the CFD model. For each cross-section, we defined the different layers of the vessel wall, incorporating the internal elastic lamina (IEL) as a $20\mu m$ offset from the endothelium (Prati et al., 2010). The remaining wall thickness corresponds to the media layer, as we are not modeling the adventitia due to its negligible effects on atherosclerosis. However, the thicknesses of these membranes, essential for accurately calculating plasma and LDL filtration, were incorporated into the relevant equations to ensure realistic modeling of the filtration process. The geometrical data of each layer is summarized in Table 2.

In this model, WSS is utilized to compute the concentration of oxLDL along the arterial wall. The mass transport process initiates with the filtration of plasma through the endothelium. This filtration is driven by changes in endothelial porosity, which are directly correlated with the magnitude of WSS. Decreased WSS leads to altered permeability, facilitating the translocation of plasma and substances such as LDL, into the intima. Once LDL molecules penetrate the arterial wall, they undergo oxidation, transforming into oxLDL. Inside the arterial wall, oxLDL molecules continue to move due to both convective effects, caused by plasma filtration, and diffusive effects, driven by concentration gradients within the wall. This establishes the concentration gradient of oxLDL across the intima layer, setting the stage for further biological interactions within the arterial wall. All the parameters needed for the computation of the mass transport model are summarized in Table 3.

1.1.1 Plasma filtration

Plasma filtration along the wall was modeled using Darcy's law (Eq. 3), which computes the filtration velocity ($\mathbf{u_p}$) based on the permeability of the medium (K), the dynamic viscosity of plasma (μ_p), and the pressure drop across the medium (∇P).

We set the initial plasma velocity filtrating across the endothelium and the pressure at the EEL as boundary conditions.

The solvent flow across a semipermeable membrane depends on the difference in the hydrostatic pressure (ΔP) and the osmotic pressure (Π), as defined by Starling's law (Eq. S1):

$$J_v = L_p(\Delta P - \Pi) \tag{S1}$$

However, according to Kedem-Katchalsky (Kedem and Katchalsky, 1958), biological membranes are not completely semipermeable and allow solute passage. Therefore, it is necessary to include a membrane reflection coefficient (σ_f), representing the membrane's resistance to solute passage. This can also be expressed in terms of the efficacy of the filtration ϵ, where $\epsilon = 1 - \sigma_f$. Thus, Equation S1 can be rewritten for biological membranes as follows:

$$J_v = L_p(\Delta P - \sigma_f \Pi) = L_p(\Delta P - (1 - \epsilon)\Pi) \tag{S2}$$

Some authors have considered the osmotic pressure negligible compared to the hydrostatic pressure (Tedgui and Lever, 1984), so Equation S2 can be simplified to Equation 4.

We determined the plasma filtration flow across the endothelium based on the three-pore theory (Michel and Curry, 1999). This theory considers different pathways for substances such as plasma or LDL to cross the endothelium: normal junctions (nj), leaky junctions (lj), and vesicular pathways (vp) (see Fig. 2). Normal junctions are the tight spaces between healthy endothelial cells and usually have an approximate size of $2 - 4$ nm. However, in areas with oscillatory or disturbed blood flow, the mechano-sensing of endothelial cells is altered. This leads to a change in their shape, making them more circular and forming leaky junctions, which are believed to have an approximate size of 20 nm.

Then, we can apply the three-pore model to Equation 4 and compute the total flow across the endothelium as the sum of the flow across each pore type (Eq. 5).

We considered the plasma flow across the vesicular pathways to be negligible (Olgac et al., 2008), so Equation 5 can be expressed as in Equation 6, where $L_{p,nj}$ and $L_{p,lj}$ represent the hydraulic conductivities of the membrane attributed to normal junctions and leaky junctions, respectively. Pressure drop at the endothelium was obtained from the experimental study conducted by Tedgui and Lever (1984), which analyzed the pressure drop in vessels with and without endothelium. The study observed that the presence of endothelium resulted in a pressure drop of 21 mmHg.

We can consider that the plasma flow across the vesicular pathways is negligible (Olgac et al., 2008). The flux across the normal junctions can be defined as follows:

$$J_{v,nj} = L_{p,nj} \Delta P_{end} \tag{S3}$$

where $L_{p,nj}$ is the hydraulic conductivity of the membrane due to the normal junctions.

The hydraulic permeability of the membrane, assuming it is completely formed by normal junctions ($L_{p,snj}$), was obtained from Tedgui and Lever (1984), where several experiments were conducted on arteries with and without endothelium to determine the hydraulic conductivity of the arterial wall and the endothelium. However, only a portion of the membrane is composed of normal junctions, so the definition of a partition coefficient (Φ_{nj}) is needed:

$$\Phi_{nj} = \frac{A_{nj}}{A_T} \tag{S4}$$

where A_{nj} is the area occupied by the normal junctions and A_T is the total area. Φ_{nj} depends on the state of the endothelium and can be related to the portion of lj:

$$\Phi_{nj} = 1 - \Phi_{lj} \tag{S5}$$

Then, $L_{p,nj}$ can be written as:

$$L_{p,nj} = \Phi_{nj} L_{p,snj} \tag{S6}$$

The flux across the leaky junctions can be defined as follows:

$$J_{v,lj} = L_{p,lj} \Delta P_{end} \tag{S7}$$

where $L_{p,lj}$ is the hydraulic conductivity of the membrane due to the leaky junctions. As with the previous parameter, this can be expressed as the hydraulic conductivity of the membrane if it were completely made of leaky junctions. However, in this case, minored by a partition coefficient representing the portion of the membrane occupied by leaky junctions (Φ_{lj}):

$$\Phi_{lj} = \frac{A_{lj}}{A_T} \tag{S8}$$

where A_{lj} is the area occupied by the leaky junctions. So, $L_{p,lj}$ can be written

as:

$$L_{p,lj} = \Phi_{lj} L_{p,slj} \tag{S9}$$

The distribution of leaky junctions was previously studied in Michel and Curry (1999) and Olgac et al. (2008). They proposed models assuming a homogeneous distribution of leaky cells and leaky junctions separated by a distance ξ. Following this description, the area of a leaky cell can be written as the area of a circle (πR_{cell}^2) and the area of a leaky junction as the area of a ring:

$$A_{lj} = \pi(R_{cell} + 2w_l)^2 - \pi R_{cell}^2 = \pi(R_{cell}^2 + 4w_l^2 + 4R_{cell}w_l - R_{cell}^2) \tag{S10}$$

Since $w_l = 20\ nm$ whereas $R_{cell} = 15\ \mu m$, the term w_l^2 can be neglected.

$$A_{lj} = \pi(4R_{cell}w_l) \tag{S11}$$

Therefore, Equation S8 can be rewritten as follows:

$$\Phi_{lj} = \frac{A_{lj}}{A_T} = \frac{4R_{cell}w_l}{\xi^2} \tag{S12}$$

Olgac et al. (2008) established a relationship between the portion of leaky cells and the portion of leaky junctions as a function of WSS. They hypothesized that leaky junctions originate from leaky cells, which are circular-shaped cells undergoing apoptosis. However, other circular-shaped cells, such as mitotic cells (MiC) may also coexist.

To model this change of shape, Levesque et al. (1986) proposed an experimental relationship based on a shape index (SI). This variable indicates the shape of the cell (Eq. S13) and can take values from 0 to 1, where 0 represents a completely spread cell and 1 represents a perfectly circular cell.

$$SI = \frac{4\pi \cdot \text{Area}}{\text{Perimeter}^2} \tag{S13}$$

Here, the area and perimeter refer to a single cell.

Using experimental data from studies on the alignment of endothelial cells in response to shear stress (Chien, 2008; Sakamoto et al., 2004; Suciu, 1997; Levesque et al., 1986), Olgac et al. (2008) derived a phenomenological equation to relate SI and WSS:

$$SI = 0.380e^{-0.790 \cdot WSS} + 0.225e^{-0.043 \cdot WSS} \tag{S14}$$

In their study, Olgac et al. (2008) also developed a phenomenological relation to estimate the number of MiCs based on the experimental data from Chien (2008), that investigated the number of MiCs in regions with specific shape indices per unit area ($A_{unit} = 0.64$ mm^2):

$$MiC = 0.003797e^{14.75 \cdot SI} \tag{S15}$$

This number of MiCs is based on the SI, thus including all circular-shaped cells in the endothelium, both MiCs and leaky cells (LCs). Therefore, Olgac et al. (2008) proposed another phenomenological relation to determine only the number of LCs from the number of MiCs:

$$LC = 0.307 + 0.805 \cdot MiC \tag{S16}$$

With all these phenomenological relations, Olgac et al. (2008) proposed a new relation to obtain the portion of endothelium occupied by LCs (Φ_{LC}):

$$\Phi_{LC} = \frac{LC \cdot \pi \cdot R_{cell}^2}{A_{unit}} \tag{S17}$$

So, we can now write Φ_{lj} as a function of Φ_{nj}:

$$\frac{\Phi_{lj}}{\Phi_{LC}} = \frac{\frac{4R_{cell}w_l}{\xi^2}}{\frac{R_{cell}^2}{\xi^2}} \tag{S18}$$

$$\Phi_{lj} = \frac{4w_l}{R_{cell}} \cdot \Phi_{LC} \tag{S19}$$

With this, all the parameters needed for the computation of plasma filtration across the wall are completely defined.

1.1.2 LDL filtration

The flux of LDL in the wall (N_{LDL}) was computed through Equation 7, in which the first and second terms are due to diffusion and convection of LDL, respectively.

The temporal evolution of LDL concentration in the artery wall was modeled using the convection-diffusion-reaction equations (Eq. 8).

As boundary conditions for the filtration process of the LDL we defined a specific flux at the endothelium based on the three-pore theory (Michel and Curry, 1999) combined with the Kedem-Katchalsky equations (Kedem and

Katchalsky, 1958), and a determined LDL concentration at the EEL.

Kedem and Katchalsky (1958) also proposed an expression for the flux of solutes across a membrane, especially emphasizing that the total solute flux is the sum of a diffusive and a convective flow (Eq. 9). We used a variant of Equation 9 that has also been used by other authors (Patlak et al., 1963; Tarbell, 2003), expressing Equation 9 in terms of the Peclet number (Eq. 10).

Due to the size of LDL molecules (20 nm), the transport across normal junctions is zero. In addition, some studies have shown that the transport of LDL across the vesicular pathways is approximately 10% of the transport across the leaky junctions (Cancel et al., 2007) (Eqs. 12, 13, 14).

From the Fick law, we can say the following related to the diffusive flux of a solute across a membrane:

$$J_A = -D_{AB} \cdot \frac{dC_A}{dx} = \frac{-D_{AB} \cdot \Phi}{L} \cdot (C_{A,r} - C_{A,l}) \tag{S20}$$

where J_A is the flux of the solute A, D_{AB} is the diffusivity of A in B, Φ is the portion of area occupied by pores, L is the thickness of the membrane and $C_{A,r}$ and $C_{A,l}$ are the concentrations of A in the right and the left side of the membrane respectively. According to this, we can define $D_{AB} \cdot \Phi$ as the permeability of the membrane P $[m^2/s]$ and $\frac{D_{AB} \cdot \Phi}{L}$ as the 'permeanza' or permeability per unit thickness $[m/s]$. Then, we can translate this to the leaky junctions:

$$P_{L,lj} = \frac{D_{lj}}{\Delta x} \Phi_{lj} \tag{S21}$$

where D_{lj} is the diffusion coefficient of the LDL in the leaky junctions, Δx is the length of the leaky junctions (equal to the thickness of the endothelium) and Φ_{lj} is the portion of leaky junctions computed before.

An experimental relation for the diffusion coefficient of LDL in the leaky junctions was proposed by Curry (1983) and has been widely used until today by several authors (Karner et al., 2001; Olgac et al., 2008; Hernández-López et al., 2021). This expression accounts for the limitation of an LDL molecule when passing across a slit pore, due to both its size (ε_1) and the collisions with the wall of the pore (ε_2).

$$\frac{D_{lj}}{D_{free}} = \varepsilon = \varepsilon_1 \cdot \varepsilon_2 \tag{S22}$$

$$\varepsilon = (1 - \alpha_{lj})(1 - 1.004\alpha_{lj} + 0.418\alpha_{lj}^3 - 0.16\alpha_{lj}^5) \tag{S23}$$

where D_{free} represents the diffusion coefficient of LDL in a free medium and

$\alpha_{lj} = \frac{r_{LDL}}{w_l}$ is a relation between the LDL molecule radius and the width of the slit.

In addition, we can assume that the concentration of LDL in the lumen is much higher than in the wall, so $\Delta C = C_l - \cancel{C_w} = C_l$. Then, Equation 13 can be rewritten as follows:

$$J_s = 1.1(P_{L,lj} C_l Z_{i,lj} + J_{v,lj}(1 - \sigma_{f,lj}) C_l) \tag{S24}$$

where $Z_i = \frac{Pe_{lj}}{e^{Pe_{lj}} - 1}$ is an intrinsic saturation factor of the diffusive term in the leaky junctions. Since C_l is present in both terms of the equation, we can simplify it as follows:

$$J_s = 1.1 P_{app,lj} C_l \tag{S25}$$

where $P_{app,lj} = P_{L,lj} Z_{i,lj} + J_{v,lj}(1 - \sigma_{f,lj})$ is the apparent permeability of the medium.

Taking all this into account, we computed the oxLDL concentration solving in 2D Equations (7 and 8) for the arterial wall using a time-dependent simulation.

References

Cancel, L. M., Fitting, A., and Tarbell, J. M. (2007). In vitro study of ldl transport under pressurized (convective) conditions. *American Journal of Physiology-Heart and Circulatory Physiology* 293, H126–H132

Chien, S. (2008). Effects of disturbed flow on endothelial cells. *Annals of Biomedical Engineering* 36, 554–562

Curry, F. (1983). Mechanics and thermodynamics of transcapillary exchange. *Handbook of Physiology. The Cardiovascular System. Microcirculation, Section 2* , 309–374

Hernández-López, P., Cilla, M., Martínez, M., and Peña, E. (2021). Effects of the haemodynamic stimulus on the location of carotid plaques based on a patient-specific mechanobiological plaque atheroma formation model. *Frontiers in Bioengineering and Biotechnology* 9, 690685

Karner, G., Perktold, K., and Zehentner, H. P. (2001). Computational modeling of macromolecule transport in the arterial wall. *Computer Methods in Biomechanics and Biomedical Engineering* 4, 491–504

Kedem, O. and Katchalsky, A. (1958). Thermodynamic analysis of the permeability of biological membranes to non-electrolytes. *Biochimica et Biophysica Acta* 27, 229–246

Levesque, M. J., Liepsch, D., Moravec, S., and Nerem, R. M. (1986). Correlation of endothelial cell shape and wall shear stress in a stenosed dog aorta. *Arteriosclerosis* 6, 220–229

Michel, C. and Curry, F. (1999). Microvascular permeability. *Physiological Reviews* 79, 703–761

Olgac, U., Kurtcuoglu, V., and Poulikakos, D. (2008). Computational modeling of coupled blood-wall mass transport of ldl: effects of local wall shear stress. *American Journal of Physiology-Heart and Circulatory Physiology* 294, H909–H919

Patlak, C., Goldstein, D., and Hoffman, J. (1963). The flow of solute and solvent across a two-membrane system. *Journal of Theoretical Biology* 5, 426–442

Prati, F., Regar, E., Mintz, G. S., Arbustini, E., Di Mario, C., Jang, I.-K., et al. (2010). Expert review document on methodology, terminology, and clinical applications of optical coherence tomography: physical principles, methodology of image acquisition, and clinical application for assessment of coronary arteries and atherosclerosis. *European Heart Journal* 31, 401–415

Sakamoto, N., Ohashi, T., and Sato, M. (2004). Effect of shear stress on permeability of vascular endothelial monolayer cocultured with smooth muscle cells. *JSME International Journal Series C Mechanical Systems, Machine Elements and Manufacturing* 47, 992–999

Suciu, A. (1997). *Effects of external forces on endothelial cells*. Tech. rep., EPFL

Tarbell, J. M. (2003). Mass transport in arteries and the localization of atherosclerosis. *Annual Review of Biomedical Engineering* 5, 79–118

Tedgui, A. and Lever, M. (1984). Filtration through damaged and undamaged rabbit thoracic aorta. *American Journal of Physiology-Heart and Circulatory Physiology* 247, H784–H791
